# Development and validation of predictive models for unplanned hospitalization in the Basque Country: analyzing the variability of non-deterministic algorithms

**DOI:** 10.1186/s12911-023-02226-z

**Published:** 2023-08-05

**Authors:** Alexander Olza, Eduardo Millán, María Xosé Rodríguez-Álvarez

**Affiliations:** 1 Basque Center for Applied Mathematics (BCAM), Bilbao, Spain; 2 Network for Research on Chronicity, Primary Care, and Health Promotion (RICAPPS), Barakaldo, Spain; 3 General Directorate for Healthcare, Osakidetza Basque Health Service, Vitoria, Spain; 4 Kronikgune Institute for Health Services Research, Vitoria, Spain; 5 CINBIO, Department of Statistics and OR, Universidade de Vigo, Vigo, Spain; 6 CITMAga Center for Mathematical Research and Technology of Galicia, Santiago de Compostela, Spain

**Keywords:** Hospitalization, Non-deterministic algorithms, Predictive models

## Abstract

**Background:**

The progressive ageing in developed countries entails an increase in multimorbidity. Population-wide predictive models for adverse health outcomes are crucial to address these growing healthcare needs. The main objective of this study is to develop and validate a population-based prognostic model to predict the probability of unplanned hospitalization in the Basque Country, through comparing the performance of a logistic regression model and three families of machine learning models.

**Methods:**

Using age, sex, diagnoses and drug prescriptions previously transformed by the Johns Hopkins Adjusted Clinical Groups (ACG) System, we predict the probability of unplanned hospitalization in the Basque Country (2.2 million inhabitants) using several techniques. When dealing with non-deterministic algorithms, comparing a single model per technique is not enough to choose the best approach. Thus, we conduct 40 experiments per family of models - Random Forest, Gradient Boosting Decision Trees and Multilayer Perceptrons - and compare them to Logistic Regression. Models’ performance are compared both population-wide and for the 20,000 patients with the highest predicted probabilities, as a hypothetical high-risk group to intervene on.

**Results:**

The best-performing technique is Multilayer Perceptron, followed by Gradient Boosting Decision Trees, Logistic Regression and Random Forest. Multilayer Perceptrons also have the lowest variability, around an order of magnitude less than Random Forests. Median area under the ROC curve, average precision and positive predictive value range from 0.789 to 0.802, 0.237 to 0.257 and 0.485 to 0.511, respectively. For Brier Score the median values are 0.048 for all techniques. There is some overlap between the algorithms. For instance, Gradient Boosting Decision Trees perform better than Logistic Regression more than 75% of the time, but not always.

**Conclusions:**

All models have good global performance. The only family that is consistently superior to Logistic Regression is Multilayer Perceptron, showing a very reliable performance with the lowest variability.

**Supplementary Information:**

The online version contains supplementary material available at 10.1186/s12911-023-02226-z.

## Introduction

The progressive ageing and longer life expectancy in developed countries [1] is leading to an increase in the number of people with chronic pathologies, fragility, functional deterioration and, in short, high care needs. This situation forces adapting the current type of healthcare offered – initially focused on acute intervention – towards a proactive system capable of anticipating people’s health problems and offering person-centered care [2]. Hence, there has been a great effort aimed at identifying populations with high future health needs in order to implement individualized preventive measures [3]. In this setting, predictive and segmentation tools facilitate population-wide stratification to achieve both improved quality of life and more efficient resource management.

The Basque Country is an autonomous region of Spain with around 2.2 million inhabitants, 23% of whose population is over 65 years old [4]. In anticipation of this ageing problem, the Autonomous Government incorporated population risk prediction tools as a fundamental element within its strategy to tackle the challenge of chronicity [5]. The current approach is a predictive model of healthcare costs based on variables transformed by the Adjusted Clinical Groups (ACG) system [6] and validated for the Basque population [7]. The model allows the patients to be ranked and segmented according to the predicted healthcare expenditures in the following 12 months.

Although the model’s discriminatory capacity to detect the basque population with the highest healthcare costs is high [7], the difficulty of its clinical interpretation – healthcare costs is acting as a proxy for healthcare needs – makes it necessary to consider other tools for predicting adverse events that are (a) relevant for the patient and their families and for clinicians and healthcare managers, (b) preventable in a significant percentage if care pathways are applied in time, or, (c) if they are not preventable, the experience of the transition through this process/event is the best possible. In the case of unplanned hospitalization, hospital discharges are critical moments regarding continuity of care and they can have negative impacts both emotionally and on quality of care and outcomes of patients, providers’ satisfaction and health system efficiency. Indeed, many health systems are already using population-based predictive models of events such as the risk of hospital admission or death [8–12].

For the development of predictive models, traditional models such as logistic regression are the norm, yet new mathematical approaches, especially machine learning methods, are being explored [12]. There is extensive literature comparing the performance of different statistical and machine learning predictive models for adverse health outcomes on specific data sets [13–15]. Often, the intent behind these examples is to choose the most appropriate modelling technique for the task at hand. However, when dealing with non-deterministic algorithms, the comparison of a single model per technique is not enough to conclude that one algorithm is superior to another.

The main objective of this study is (a) to develop and externally validate a population-based prognostic model to predict the probability of unplanned hospitalization in the Basque Country through (b) comparing the performance of a logistic regression model and three families of machine learning models. For the machine learning techniques, we evaluate the variability of models’ performance to be able to make an informed decision on the best approach for our specific problem.

## Materials and Methods

### Data and overall methodology

 **Setting:** Cross-sectional study conducted in the Basque Country, an autonomous region of Spain with around 2.2 million inhabitants. The Basque Health Service (Osakidetza) provides universal and free health care services – except for a co-payment to purchase prescribed drugs – to the Basque population. Osakidetza is divided into 13 Integrated Healthcare Organisations (IHO). Each IHO manages both primary and hospital care in their catchment population.

**Fig. 1:**
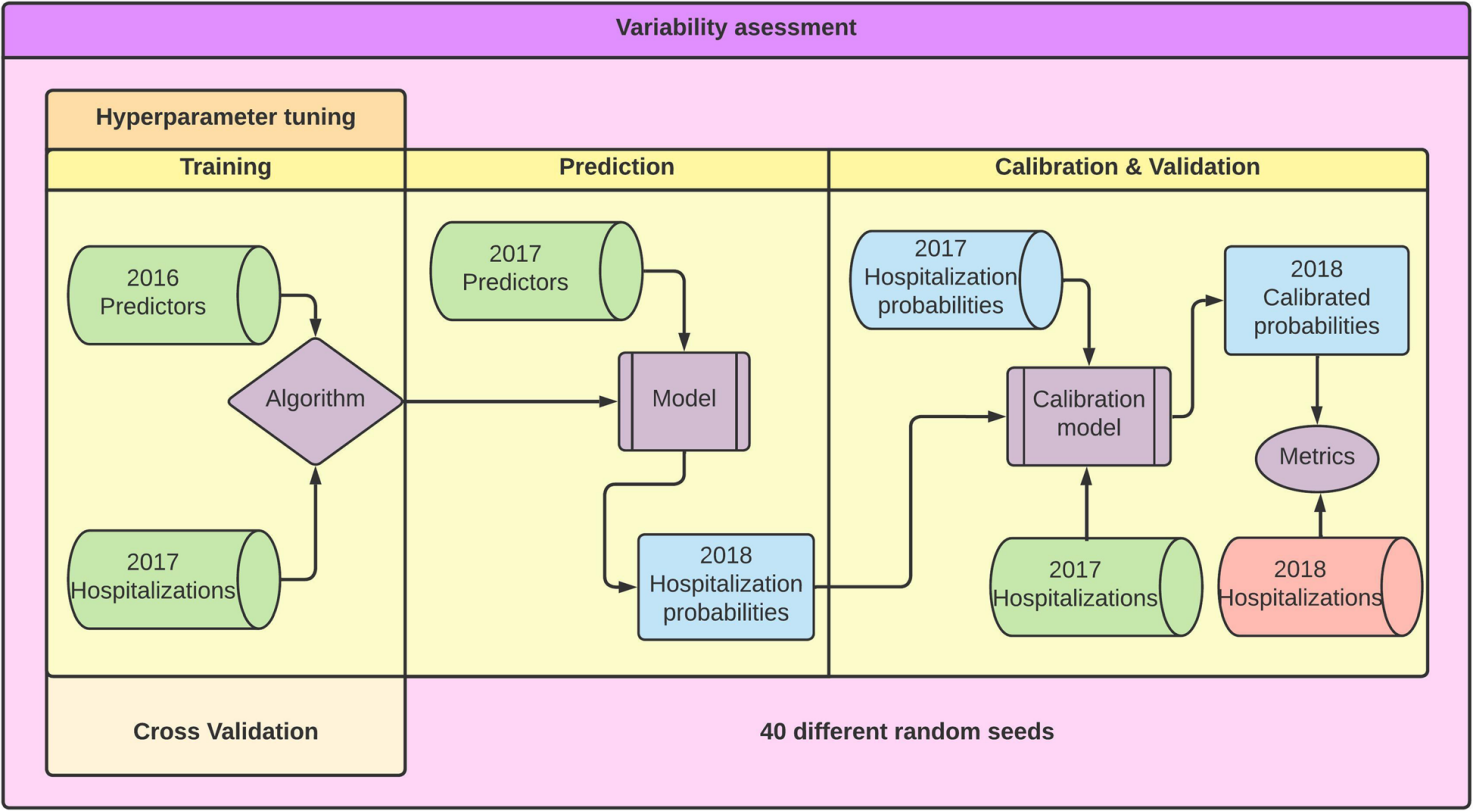
Flux diagram of our methodology, divided in three stages and repeated 40 times per non-deterministic algorithm. Necessary input data is inside green cylinders, predictions are in blue and red denotes data that will not be available at the time of application

**Study population:** Predictive models were developed based on all patients enrolled in Osakidetza in 2016 (N = 2240526). The external validation sample comprised all patients enrolled in Osakidetza in 2017 (N = 2241276).

**Study variables:** Variables included in the models were those suggested by The Johns Hopkins ACG System Version 11.1 Technical Reference Guide, including age and gender, Adjusted Clinical Groups (ACG, mutually exclusive isomorbidity clusters) that represent the most resource intensive morbidity groups; Expanded Diagnosis Clusters (EDC, e.g. Glaucoma, Endometriosis) for which the evidence linking health care to outcomes is strong; and pharmacy-based markers Rx-MGs (Rx-MG, e.g. Infections/Acute Minor, General Signs and Symptoms/Pain). ACGs, EDCs and Rx-MGs were computed by the Johns Hopkins ACG software, a case-mix system that processes all the codified diagnoses and dispensed drugs in the electronic health record (EHR) to characterize the population through ACGs, EDCs and Rx-MGs. All the variables are binary except age (categorized) and ACG (categorical). See Appendix for a brief description and Additional File for the full list of variables.

Although individuals in the same ACG category share clinical characteristics and similar expected needs for healthcare resources, ACGs capacity to predict the risk of hospitalization can be further improved by adding other variables such as EDCs or Rx-MGs (Appendix, Table 3).

The response variable was whether the patient had any unplanned admission in the following year (2017 during development, 2018 during validation). We excluded all unplanned admissions related to traumatic injuries, birth and delivery. We considered them to be either unpredictable or non-avoidable. To define unplanned admissions we followed the algorithm developed by the Centers for Medicare and Medicaid Services [16, 17]. In this way, we avoided the administrative labelling of admissions to focus on the clinical perspective. Regarding the construction process of the ACGs, EDCs and Rx-MGs, we only considered diagnoses with any activity during the corresponding year. This includes illnesses with diagnoses recorded in the same year, and previous diagnoses with follow-up activity such as treatment or medical visits.

**Statistical analysis:** We predicted the probability of an unplanned admission using Logistic Regression (LR), Random Forest (RF), Gradient Boosting Decision Trees (GBDT) and Multilayer Perceptrons (MLP). The overall methodology is described in Fig. 1. For all techniques, we trained the models with predictors from 2016 and hospitalizations from 2017 (Fig. 1, left block). We then fed the best model with predictors from 2016 and from 2017 to obtain predictions for 2017 and for 2018 respectively (Fig. 1, central block). We built a separate calibration model, and trained it with the predictions for 2017 and the corresponding observed events. This model then received the predicted probabilities for 2018 as input, and returned the final calibrated probabilities. We externally validated the models using the calibrated probabilities and the observed events in 2018 (Fig. 1, right block). For MLP, RF and GBDT we repeated the whole process 40 times due to its non-deterministic nature. We expand on the statistical analysis in the following sections, where we also describe the metrics used to evaluate models’ performance.

### Variability assessment

Except LR, the other methods considered in this study are non-deterministic, in the sense that they can lead to different models for the same data-set. This is due to several sources of randomness:

- The tree ensembles (RF and GBDT) require the classes to be balanced before training, whereas the LR and the MLPs are able to use the whole data-set. Given the dimensionality of our data, we achieved balanced classes using random undersampling.
- RF, GBDT and MLP have multiple hyperparameters that need to be selected. For computational reasons, hyperparameter tuning usually explores only a subspace of the grid of possible values, choosing the best model within that sample. Commonly, these grid points are selected at random. Different samples of grid points may give rise to diverse selected models.
- RF is intrinsically non-deterministic.

Considering all of this, different realizations of the entire process may lead to diverse rankings of the techniques. We studied the variability of the three non-deterministic algorithms using 40 different random seeds. This controls all sources of randomness, namely class balancing, hyperparameter tuning and intrinsic randomness.

For the tree ensembles, we tested 30 random hyperparameters combinations per seed, using 3-fold Cross-Validation (CV) and optimizing the validation accuracy score, which is the default evaluation metric in scikit-learn. We note that, since we are using balanced classes, the accuracy is a valid measure. For the MLP family, we tested 100 hyperparameters combinations per seed with a training horizon of at most 30 epochs, once again with 3-fold CV, but this time optimizing the validation loss (binary cross-entropy). Once the tuning was complete, we retrained the MLP models with early-stopping to prevent overfitting, interrupting the training process as soon as the validation loss stopped decreasing for 5 consecutive epochs, and keeping the best weights for the final models. We built these models using Keras (Tensorflow).

A limit of 80 hours was set for each experiment, aborting those that did not finish within that time frame. This condition may introduce a selection bias when assessing the performance of each technique. However, if the interest is a practical application, excessive computational demand may also be a reason to discard a model.

### Post-calibration

A probabilistic classifier is referred to as calibrated when its predictions are reliable estimates of the true probability for each class. By contrast, an uncalibrated classifier can only be trusted in its ability to order individuals according to risk. When probability estimates are used for clinical decision-making, assessing model calibration becomes essential. This allows population-wide stratification based on probability ranges, which could be displayed in the EHR.

It is well known that tree ensembles produce distorted class probability distributions [18] and need posterior calibration, commonly achieved by means of isotonic regression. This approach yields a non-decreasing step function that gives calibrated predictions. Its non-decreasing property avoids permuting the predicted probabilities, leaving the discriminatory metrics unaffected. However, the step function homogenizes the predictions into large groups, hindering stratification. To prevent that, we used Piecewise Cubic Hermite Interpolating Polynomials (PCHIP) after isotonic regression to obtain smooth calibrated probabilities [19]. This combination of techniques is non-parametric, preserves discrimination, and produces a smooth distribution of predicted probabilities, allowing flexible stratification.

### Performance metrics

The intended use of the models developed in this work is both population-wide prediction and high-risk patient identification subject to resource management consideration. Therefore, we used both threshold-independent and threshold-dependent metrics to evaluate the models.

The first type of metrics do not depend on a pre-specified decision criterion (i.e., a pre-specified value of the predicted probability above which we consider that the patient will likely be hospitalized the following year). We chose the area under the ROC curve (AUC) and the Average Precision (AP) to evaluate this aspect of performance, as well as the corresponding ROC and Precision-Recall (PR) curves. In a coin-toss model, the value of AP is equal to the fraction of individuals of the positive class (in our case the fraction of patients that had an unplanned hospitalization). In such random model, the AUC would be 0.5. For a perfect model, both AP and AUC would be 1. In unbalanced data-sets, small differences between models in the ROC space translate to bigger differences in the precision-recall space [20]. Thus, AP is more informative than AUC in such data-sets [21].

To emulate a clinical decision-making scenario, the ability to identify high-risk patients is best measured with metrics that depend on the classification of patients (likely to be hospitalized versus not likely). We report R@K and PPV@K, which represent the recall (sensitivity) and positive predictive value among the *K* patients with the highest predicted probabilities (high-risk group). In terms of sets and in terms of true versus predicted classes, these metrics are described in Eq. (1):

1 $$\begin{aligned} R{@}K= & {} \frac{\vert L_K\cap H\vert }{\vert H\vert }\times 100 = \frac{TP}{TP+FN}\times 100 \nonumber \\ PPV@K= & {} \frac{|L_K\cap H|}{K}\times 100 = \frac{TP}{TP+FP}\times 100 \end{aligned}$$

where $L_K$ is the list of *K* patients with the highest predicted probability, hence considered as positive, *H* is the list of patients who had unplanned hospitalizations, and the abbreviations *TP*, *FN* and *FP* stand for True Positives, False Negatives and False Positives.

Note that rather than choosing an “optimal” probability threshold for the classification, we fixed the number of positive class predictions (individuals likely to be hospitalized) at *K* to depict resource-management concerns. After the identification of high-risk individuals, such a list of patients will be delivered to the healthcare providers to find preventive interventions. In particular, we chose a list of $K=20000$ individuals, corresponding to about $10 - 15$ patients per primary care team in the Basque Country, because it is a manageable quantity to revise. We also report the results for the top 5% and 10% of patients (Appendix, Table 4).

Regarding calibration we report the Brier Score *B*. This metric quantifies the average of the squared distance between the predicted probabilities, $\hat{p}_j$, and the observed binary outcomes, $o_j$, as shown in Eq. (2), where *N* is the size of the population at the prediction/validation stage. It ranges from 0 to 1, with lower values indicating better models:

2 $$\begin{aligned} B = \frac{1}{N}\sum \limits _{j=1}^N(\hat{p}_j-o_j)^2 \end{aligned}$$

The Brier Score is the only metric affected by our post-calibration procedure. It combines the effects of calibration and discrimination, but the latter remains unchanged after isotonic regression and PCHIP smoothing. Hence, the difference in Brier Score before and after this process shows the improvement in calibration.

## Results

### Description of the study population

Among the patients enrolled in the public health system in 2017 (i.e., the population used for the validation of the models), 5.67% of them had at least one unplanned hospitalization in 2018 (5.39% for women and 5.97% for men). 22.98% of women and 17.68% of men were older than 65 (Table 1).

**Table 1:** Population at the prediction/validation stage (COPD: Chronic Obstructive Pulmonary Disease). The comorbidity data indicates the number of patients with active diagnoses in 2017, i.e. those that had any related treatment, diagnostic code or medical appointment in 2017. The true prevalences of diseases may be higher

	Women	Men
N in 2017 (%)	1144488 (51.06 %)	1096788 (48.94 %)
Hospitalized in 2018	61732 (5.39 %)	65463 (5.97 %)
Aged 0-17	189733 (16.58 %)	201982 (18.42 %)
Aged 18-64	691768 (60.44 %)	700895 (63.90 %)
Aged 65-69	67088 (5.86 %)	61850 (5.64 %)
Aged 70-79	107004 (9.35 %)	89217 (8.13 %)
Aged 80-84	46711 (4.08 %)	31114 (2.84 %)
Aged 85+	42184 (3.69 %)	11730 (1.07 %)
COPD	10863 (0.95 %)	20356 (1.86 %)
Chronic renal failure	9345 (0.82 %)	10756 (0.98 %)
Heart failure	8424 (0.74 %)	7953 (0.73 %)
Depression	21489 (1.88 %)	7856 (0.72 %)
Diabetes Mellitus	47268 (4.13 %)	58745 (5.36 %)
Hypertension	104089 (9.09 %)	100132 (9.13 %)
Ischemic heart disease	5943 (0.52 %)	15514 (1.41 %)
Low back pain	98125 (8.57 %)	72982 (6.65 %)
Osteoporosis	18597 (1.62 %)	1141 (0.10 %)
Parkinson’s disease	2212 (0.19 %)	2339 (0.21 %)
Persistent asthma	34209 (2.99 %)	31060 (2.83 %)
Rheumatoid arthritis	3878 (0.34 %)	1464 (0.13 %)
Schizophrenia & affective dis.	3226 (0.28 %)	4367 (0.40 %)
Seizure disorders	4275 (0.37 %)	4874 (0.44 %)

The most prevalent diseases were hypertension (9.09% for women and 9.13% for men) low back pain (8.57% and 6.65%), and diabetes (4.13% and 5.36%). We also inspect other relevant comorbidities (Table 1).

The population in 2016 (the one used in the development/training of the models) had a similar demographic distribution, and the percentage of patients that are in either set, but not in the intersection is 3.98% (Appendix, Table 1).

### Model performance

After 80 hours of runtime, we obtained 30 RF models, 26 GBDT models and 40 MLPs, besides the logistic regression model. The results shown below are based on these models.

**Fig. 2:**
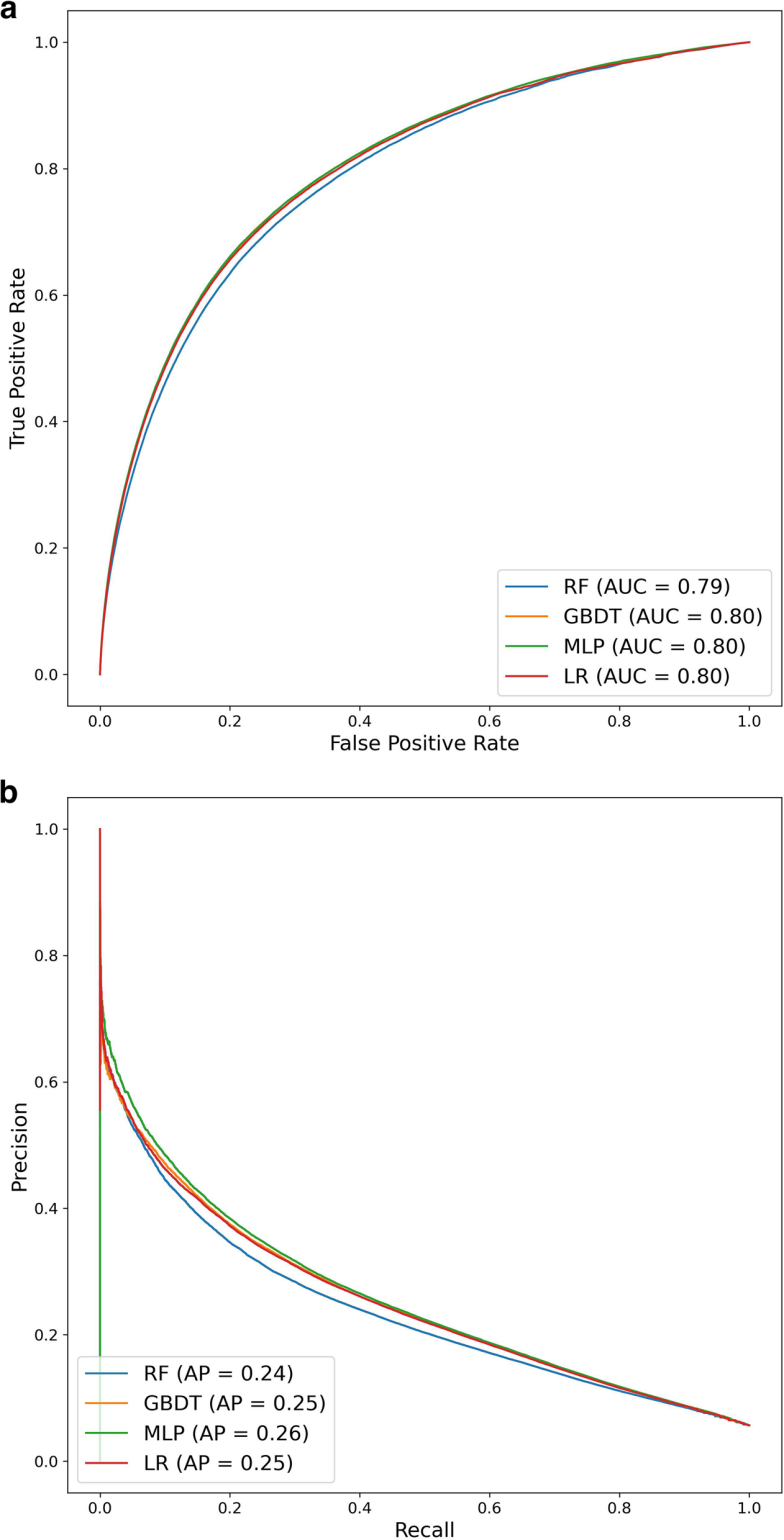
For the external validation set: ROC and PR curves for the models with median AUC and median AP, respectively. (MLP: Multi-Layer Perceptron; GBDT: Gradient-Boosting Decision Trees, LR: Logistic Regression, RF: Random Forest, AP: Average Precision)

**Overall performance of techniques:** Median AUC ranges from 0.789 to 0.802. The median AP is between 0.237 and 0.257 (coin-toss model: AP = 0.0567). Median R@20k ranges from 0.076 to 0.080. For PPV@20k, the median values are between 0.485 and 0.511 and for Brier Score the median values are of 0.048 for all techniques (Table 2). Figure 2 shows the ROC and PR curves for one model per family. The ROC curves almost overlap and, as expected under class imbalance, the differences become visible in the PR curves.

**Table 2:** For the external validation set: Summary of the performance metrics in each family of models (quartiles and standard deviation). The techniques are ordered from the best to the worst performance. (MLP: Multi-Layer Perceptron; GBDT: Gradient-Boosting Decision Trees, LR: Logistic Regression, RF: Random Forest, AP: Average Precision, R@20k and PPV@20k: Recall and Positive Predictive Value for the 20000 highest-risk patients)

	25%	50%	75%	std
**AUC**				
MLP	0.8017	0.8019	0.8020	1.89e-04
GBDT	0.8010	0.8011	0.8012	2.05e-04
LR		0.7985		
RF	0.7845	0.7888	0.7889	2.94e-03
**AP**				
MLP	0.2571	0.2574	0.2575	3.04e-04
GBDT	0.2515	0.2519	0.2528	1.21e-03
LR		0.2504		
RF	0.2297	0.2368	0.2370	4.52e-03
**R@20k**				
MLP	0.0802	0.0803	0.0805	2.38e-04
GBDT	0.0781	0.0784	0.0789	7.69e-04
LR		0.0776		
RF	0.0741	0.0760	0.0762	1.47e-03
**PPV@20k**				
MLP	0.5101	0.5108	0.5117	1.51e-03
GBDT	0.4967	0.4984	0.5018	4.89e-03
LR		0.4932		
RF	0.4712	0.4835	0.4848	9.36e-03
**Brier Score**				
MLP	0.0474	0.0475	0.0475	2.25e-05
GBDT	0.0476	0.0476	0.0476	3.72e-05
LR		0.0477		
RF	0.0482	0.0482	0.0485	1.55e-04

**The best performing technique:** For all measures the best-performing technique is MLP (AUC = 0.802; AP = 0.257 and Brier Score = 0.048), followed by GBDT, LR and RF. MLPs also have the lowest variability (Table 2). For more than 50% of the MLP models, the proposed list of 20000 patients includes more than 8.0% of all patients who were admitted in 2018; 51.1% of the patients shortlisted by MLPs will indeed be admitted the next year. This percentage drops to 48.3% with RF. Given the population size, this implies 1098 additional correct class predictions with the median MLP model instead of the RF model.

**Fig. 3:**
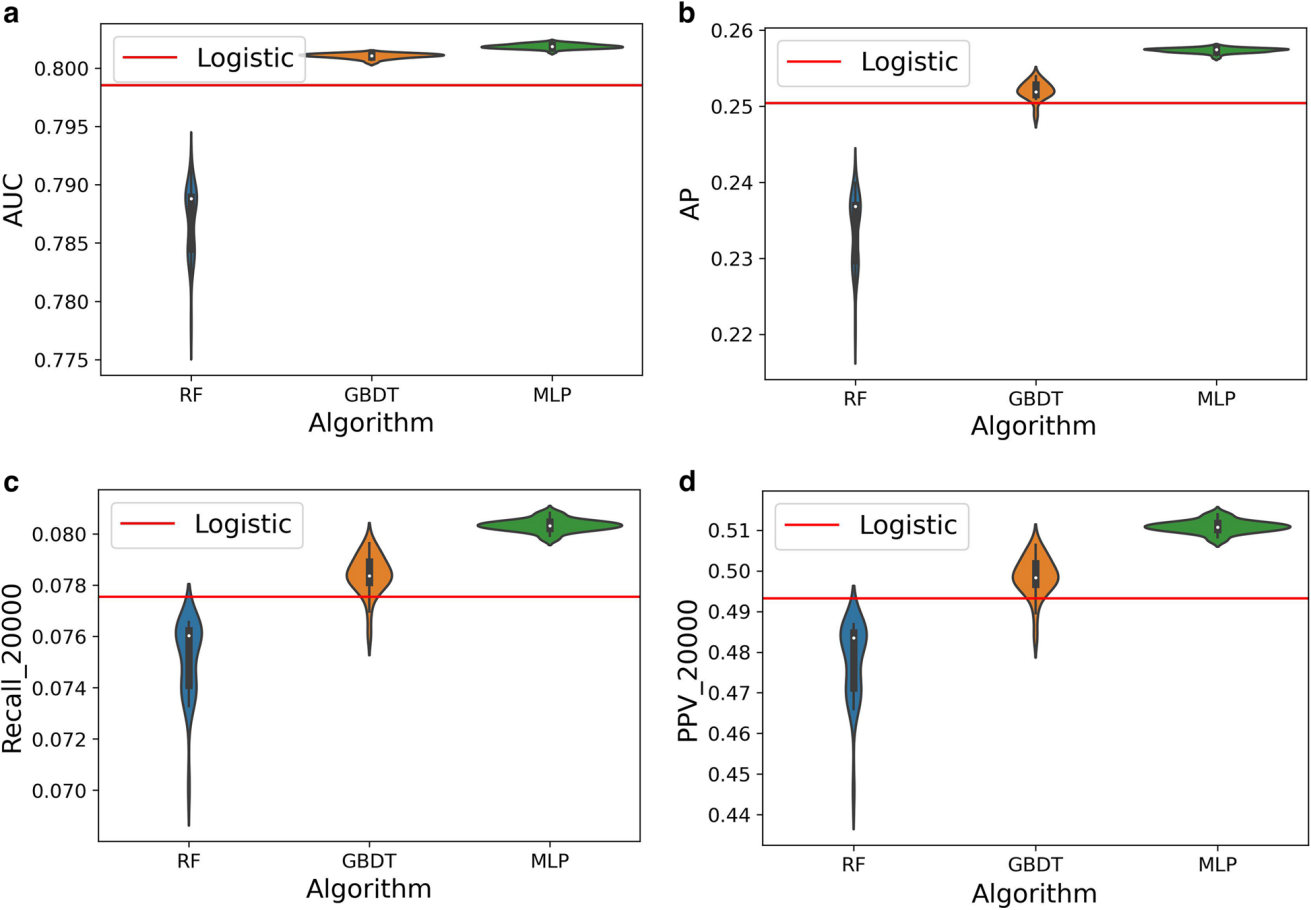
For the external validation set: Performance of the RF, GBDT and MLP families according to different discrimination metrics. The violins show the variability of each family due to stochastic effects. Red line: LR (MLP: Multi-Layer Perceptron; GBDT: Gradient-Boosting Decision Trees, LR: Logistic Regression, RF: Random Forest, AP: Average Precision, R@20k and PPV@20k: Recall and Positive Predictive Value for the 20000 highest-risk patients)

**Variability/overlapping of the algorithms:** There is some overlap between the algorithms. For instance, GBDT has better R@20k and PPV@20k than LR more than 75% of the times, but there are several experiments where GBDT performs worse than LR, and rare cases where it does worse than RF in terms of the threshold specific metrics, although not in terms of global discrimination (Fig. 3).

**Fig. 4:**
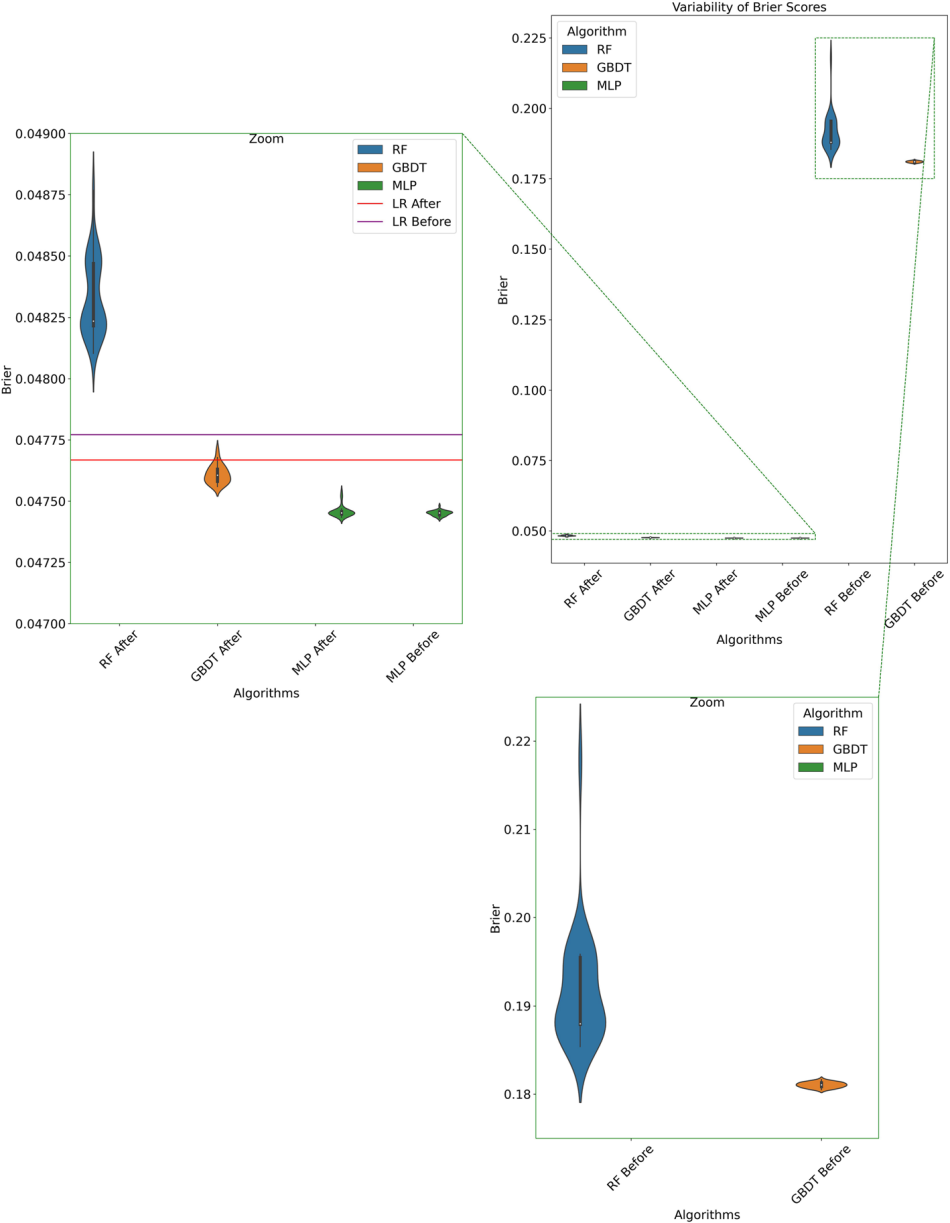
For the external validation set: Variability of the Brier Score. Purple and red lines: LR before and after post-calibration. Top-right: Comparison of all the algorithms before and after post-calibration. Bottom: Violin plots of the tree ensembles before post-calibration (high Brier Scores). Top-left: RF, GBDT and MLP after calibration, and MLP before calibration. (MLP: Multi-Layer Perceptron; GBDT: Gradient-Boosting Decision Trees, LR: Logistic Regression, RF: Random Forest)

**Fig. 5:**
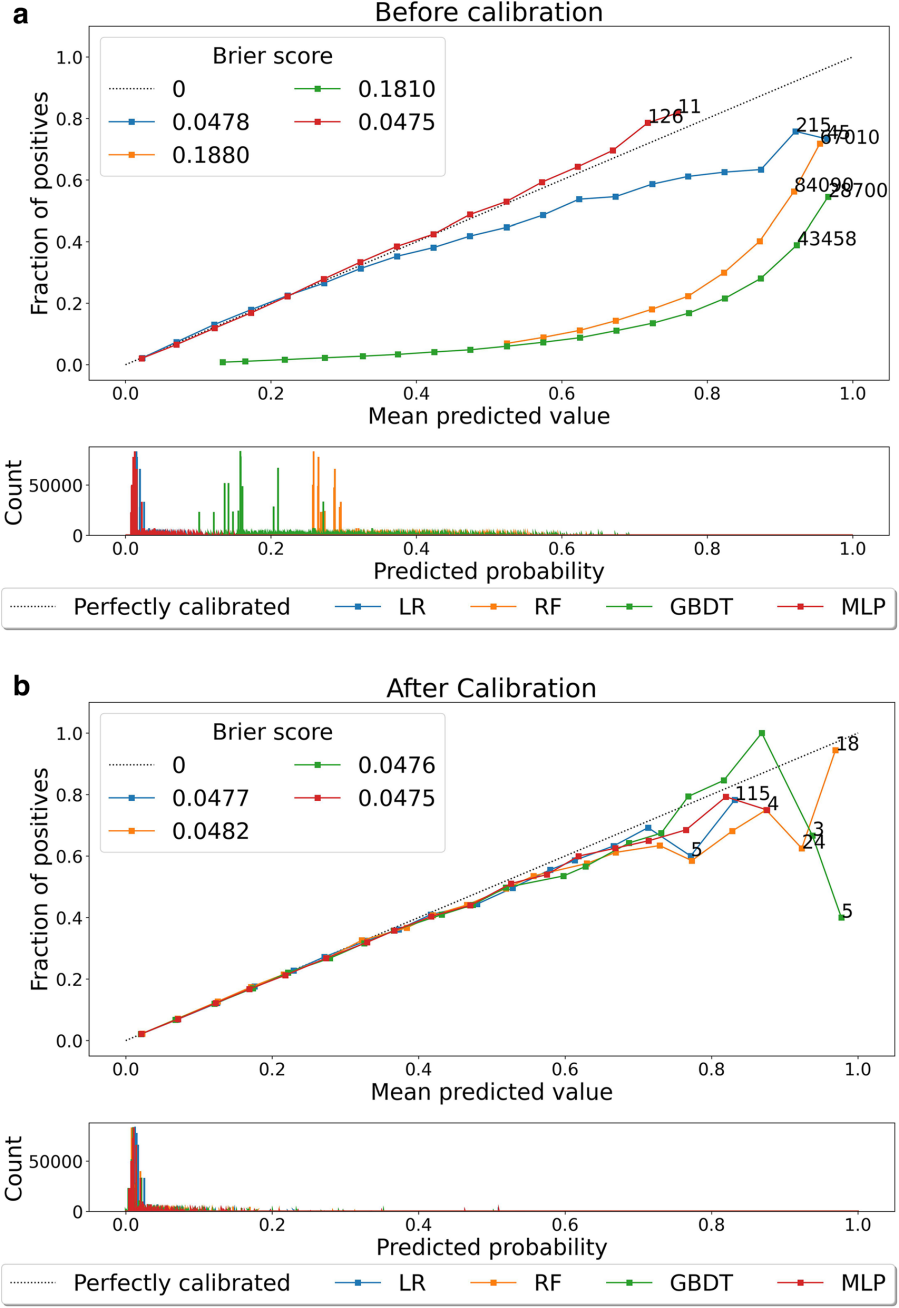
For the external validation set: Reliability diagrams (a) before, and (b) after post-calibration. The numbers indicate the amount of patients in the last two bins. The bottom part is a histogram of the predicted probabilities. (MLP: Multi-Layer Perceptron; GBDT: Gradient-Boosting Decision Trees, LR: Logistic Regression, RF: Random Forest)

**Post-calibration:** The values of the Brier Score improve after post-calibration, specially for the tree ensembles (Fig. 4). In Fig. 5 we report the reliability diagrams of the models with the median Brier Score before and after post-calibration. Again, we see a remarkable improvement in the calibration of tree ensembles.

**Overfitting:** In the training set, the median AUCs range from 0.789 to 0.805. The technique ranking in terms of R@20k and PPV@20k is different than in the external validation, placing RF on top of LR due to overfitting (Appendix, Table 2 and Supplementary Fig. 1).

### Characteristics of the risk groups

Eighty-nine percent and eighty-five percent of the patients in the high-risk groups (20000 individuals) selected by LR and the MLP with median PPV@20k are older than 65, compared to 20% in the general population (GP). The prevalence of what are considered to be complex chronic conditions is also high: 39% - 44% of identified high-risk patients have diabetes (5% in the GP), 35%-39% heart failure, 31% -33% COPD, 18%-19% ischemic heart disease and 30%-34% of them have chronic renal failure, among others. Also, the proportion of patients with multimorbidity in this high-risk population is very high, 62%-66% (Table 3).

**Table 3:** For the external validation set: Characteristics of the risk groups (20000 individuals) identified by the MLP with median performance and by LR. The comorbidity data indicates the number of patients with active diagnoses in 2017, i.e., those that had any related treatment, diagnostic code or medical appointment in 2017. The true prevalences of diseases may be higher. Pluripathologic: We follow the modified definition of pluripathology by Ollero et al. [22]: the presence of chronic diseases included in two or more of the following clinical categories: (a) ischemic heart disease (excluding acute miocardyal infarction) OR congestive heart failure; (b) chronic renal failure; (c) COPD; (d) inflammatory bowel disease OR chronic liver disease; (e) cerebrovascular disease OR dementia OR delirium; and (f) peripheral vascular disease OR diabetic retinopathy OR diabetes with complications. (COPD: Chronic Obstructive Pulmonary Disease)

	Multi-Layer Perceptron	Logistic Regression	General Population
Hospitalized in 2018	10216 (51.08 %)	9864 (49.32 %)	127195 (5.68 %)
% of Women	33.95 %	35.20 %	51.06 %
Aged 0-17	68 (0.34 %)	81 (0.41 %)	391715 (17.48 %)
Aged 18-64	2999 (14.99 %)	2200 (11.00 %)	1392663 (62.14 %)
Aged 65-69	1641 (8.21 %)	1372 (6.86 %)	128938 (5.75 %)
Aged 70-79	5634 (28.17 %)	5308 (26.54 %)	196221 (8.75 %)
Aged 80-84	3811 (19.05 %)	4498 (22.49 %)	77825 (3.47 %)
Aged 85+	5847 (29.23 %)	6541 (32.70 %)	53914 (2.41 %)
COPD	6525 (32.62 %)	6117 (30.59 %)	31219 (1.39 %)
Chronic Renal Failure	6853 (34.27 %)	5980 (29.90 %)	20101 (0.90 %)
Heart Failure	7727 (38.63 %)	6920 (34.60 %)	16377 (0.73 %)
Depression	1544 (7.72 %)	1571 (7.86 %)	29345 (1.31 %)
Diabetes Mellitus	8808 (44.04 %)	7803 (39.02 %)	106013 (4.73 %)
Hypertension	16131 (80.66 %)	14905 (74.53 %)	204221 (9.11 %)
Ischemic Heart Disease	3774 (18.87 %)	3508 (17.54 %)	21457 (0.96 %)
Low back pain	3495 (17.48 %)	3572 (17.86 %)	171107 (7.63 %)
Osteoporosis	944 (4.72 %)	944 (4.72 %)	19738 (0.88 %)
Parkinson’s disease	526 (2.63 %)	518 (2.59 %)	4551 (0.20 %)
Persistent asthma	1957 (9.79 %)	1631 (8.15 %)	65269 (2.91 %)
Rheumatoid arthritis	439 (2.19 %)	361 (1.80 %)	5342 (0.24 %)
Schizophrenia & affective dis.	627 (3.13 %)	944 (4.72 %)	7593 (0.34 %)
Seizure disorders	692 (3.46 %)	813 (4.07 %)	9149 (0.41 %)
Pluripathologic	13135 (65.67 %)	12306 (61.53 %)	61998 (2.77 %)

## Discussion

This study has achieved a double objective. First, we have obtained calibrated predictions of the risk of unplanned hospitalization for an entire population to allow stratification and planning of preventive interventions. Second, we have compared several prediction approaches, assessing the variability of non-deterministic techniques to identify those that consistently outperform others.

The importance of the application lies in its population-based nature. The use of this type of predictive models in the Basque Country would help, as a population screening tool (2.2 million inhabitants), to identify and prioritize individuals at high risk of hospitalization who could benefit from the integrated care pathways currently in use in the Basque Health Service. These integrated care pathways have been shown to reduce the risk of hospitalization in patients with complex chronic diseases and multimorbidity [23, 24], who, in fact, represent the majority of high-risk patients selected by our predictive models (see Table 3). In this way, the identification of individuals with a high risk of hospitalization would complement other preventive strategies already in place in the Basque Country for identifying high need-high risk patients. Specifically, in 2010, the Department of Health of the Basque Government launched the “Strategy to tackle the challenge of chronicity in the Basque Country” [5], aimed to re-orient the health system toward an integrated care model and a person-centred approach.

All our models have good global discrimination, with AUCs around 0.80 and APs around 0.25. Identifying the 20000 highest-risk patients is much more difficult, resulting in lists with low recall, because we impose a number of positives that is much lower than the admissions per year. Despite this low recall, the decision to identify only the 20000 most at-risk patients is based on the resource intensity that the deployment and adherence to these integrated pathways requires. According to the experience in the Basque Country, such resource-intensive integrated pathways [23, 24] – focused on (a) care coordination and communication between health providers, and (b) patient empowerment and home-based care – should not be offered to more than 10-15 patients per primary care team (there are about 1500 primary care teams in the Basque Health Service). However, the positive predictive value (around 50%) indicates that, in the screening phase by primary care physicians/teams, half of the screened patients will be hospitalized during the following year if no preventive intervention is considered. Thus, as mentioned above, the suggested lists could be very useful for clinical practice as they would help to identify patients who could benefit from adjusted patient-centered care pathways.

Regarding the evaluation of different techniques, we do not see big differences in the metrics. The only family that is always consistently superior to LR is MLP, showing a very reliable performance with the lowest variability. GBDT overall outperforms LR, but has some overlap. RF is consistently worse than LR, and also has the highest variability. In absolute terms, the MLP with median R@20k produces 704 additional correct predictions compared to LR and 1098 compared to the corresponding RF. The ACG system provides highly processed binary variables, leaving little room for machine learning techniques to excel with this specific kind of data-set.

Several factors influence whether the performance gain justifies the increased complexity of MLP versus LR. In terms of computational effort, these simple neural networks do not require considerable additional work or any special equipment. Both models could be pre-trained and implemented into a ready-to use application. Regarding interpretability, there is no doubt that LR is ahead of MLP. However, there is extensive recent work on explaining neural networks, as well as some open-source software [25–27]. Nevertheless, our predictor variables have limited interpretability by themselves due to the use of the opaque tool of ACG. Despite that, we show the 20 most important variables for selected models of each kind (Appendix, Fig. 2).

Algorithmic fairness is another concern regarding the use of machine learning in clinical practice. In this article we conclude that MLP performs better with our data in the general population, but an additional fairness analysis should be conducted before making the final decision. However, the fairness of LR must be assessed as thoroughly as that of MLP. To conduct this analysis, we must gather expert knowledge on which sensitive attributes to choose and which definition of fairness to strive for. Typical sensitive attributes are gender and socioeconomic status, but there may be many other hidden biases, and some of them may not even be measured in our variables. To begin with, both LR and MLP contain a low percentage of women in the selected risk groups (34% and 35%; Table 3), while the proportion of observed hospitalizations is only 0.6% lower for women. The possible causes of this will be researched in an upcoming work.

In terms of AUC, our results are similar to those found in the literature. Previous predictive models for hospitalization using the ACG system reported AUCs between 0.73 [28] and 0.80 [29], increasing to 0.82 with the inclusion of resource usage variables such as the number of outpatient and emergency visits, dialysis or nursing services.

Including additional variables may improve model performance, and even impact the comparison among techniques. Apart from resource usage variables, thorough information about the patients and their living conditions could be considered, accounting for social determinants of health.

The extent in overfitting is rather small for all techniques, which have similar performance in both the training and external validation sets (Appendix, Table 2 and Supplementary Fig. 1). Interestingly, GBDT sometimes has a higher performance in the training set than MLP, although the median is higher for MLP. This is due to early-stopping on the validation loss, which prevents MLPs from overfitting. In terms of the threshold-specific metrics, for the given case of $K=20000$, RF sometimes achieves the highest performance in the training set, with a median higher than LR, but its big variability makes it unreliable compared to the other families. These results mean that, without performing a variability analysis and a proper validation, there is a high chance to erroneously choose RF over LR based on risk-patient identification on the training set.

Regarding limitations of this study, given our data we have no way to account for the different healthcare use patterns of subpopulations based on gender, socioeconomic status or other sensitive attributes, which may contribute to unfairness. Additionally, we have focused only on the performance variability without analyzing any trends in the model structure. To gain insight on model complexity, it could be interesting to assess tendencies in the selected hyperparameters, such as the number of layers and neurons most commonly chosen for MLPs. We are aware that not all unplanned hospitalizations are avoidable. In this regard, the development of predictive models of the risk of a potentially avoidable hospitalization might have helped to select a more impactful population [30, 31]. However, there is a lack of consensus on which hospitalizations should be considered avoidable, and there is a high risk of leaving out a proportion of avoidable hospitalizations (sensibility concerns) [32, 33]. Also, even if hospitalization were not avoidable, we should always provide a high quality hospitalization process, including a safe and satisfying discharge and transition back to the community for the patient, caregivers and the healthcare professionals caring for them, and one way to achieve this is by putting in place all the elements of communication, empowerment and coordination that are also developed in these integrated care pathways.

For further research, we propose constructing transparent and less processed predictors to test them against the models built on top of the ACG system. Previous studies choose ACG as the recommended case-mix system among other similar tools [8]. However, to our knowledge, there is no research comparing ACG predictors against a comprehensive set of variables built on the complete list of diagnoses and prescriptions per patient.

## Conclusions

In this study, we built population-wide predictive models for unplanned hospitalization in the Basque Country, and compared several machine learning approaches considering the performance variability due to their non-deterministic nature. The best performance and lowest variability was achieved with multilayer perceptrons. The observed overlap between some of the techniques highlights the importance of a variability analysis.

### Supplementary information

**Additional file 1.** Appendix.

**Additional file 2.** Full list of variables included in the models and TRIPOD checklist: Good practices for prediction model development and validation.

## Data Availability

The data that support the findings of this study are available from Osakidetza but restrictions apply to the availability of these data, which were used under license for the current study, and so are not publicly available. Data are however available from the authors upon reasonable request and with permission of Osakidetza. The source code is available in Zenodo, with DOI 6957516. Link: https://zenodo.org/record/6957516#.ZGSnrhlBw8o Data is obtained from administrative health databases and the Osakidetza electronic health records (EHR), which integrates both primary care and hospital care including electronic pharmacological prescription. All this information is extracted by means of a business intelligence platform that links all the structured data relating to the same patient by means of pseudonymised identifiers.

## Abbreviations

ACG: Adjusted Clinical Groups
AP: Average Precision
AUC: Area Under the (Receiver Operating Characteristic) Curve
COPD: Chronic Obstructive Pulmonary Disease
EDC: Expanded Diagnostic Clusters
EHR: Electronic Health Record
GBDT: Gradient Boosting Decision Trees
LR: Logistic Regression
MLP: Multilayer Perceptron
PCHIP: Piecewise Cubic Hermite Interpolating Polynomials
RF: Random Forest
Rx-MG: Pharmacy-based markers (Rx-Defined Morbidity Groups)
