# Supplementary material for: Development and validation of predictive models for unplanned hospitalization in the Basque Country: analyzing the variability of non-deterministic algorithms
Source: BMC Med Inform Decis Mak. 2023 Aug 5;23:152. doi: 10.1186/s12911-023-02226-z (PMC10403913; doi:10.1186/s12911-023-02226-z)
Supplement: Supplementary file 1 — Additional file 1. Appendix. [file 12911_2023_2226_MOESM1_ESM.zip › appendix.pdf]

Appendix

Comparison between development and validation data

See Table 1.

	Development (2016-2017)	Validation (2017-2018)
Hospitalized in Year 2	127195 (5.68 %)	123754 (5.52 %)
% of women	51.06 %	51.03 %
Aged 0-17	391715 (17.48 %)	392511 (17.52 %)
Aged 18-64	1392663 (62.14 %)	1399536 (62.46 %)
Aged 65-69	128938 (5.75 %)	127741 (5.70 %)
Aged 70-79	196221 (8.75 %)	191509 (8.55 %)
Aged 80-84	77825 (3.47 %)	80314 (3.58 %)
Aged 85+	53914 (2.41 %)	48915 (2.18 %)
COPD	31219 (1.39 %)	31533 (1.41 %)
Chronic Renal Failure	20101 (0.90 %)	18987 (0.85 %)
Heart Failure	16377 (0.73 %)	15687 (0.70 %)
Depression	29345 (1.31 %)	30055 (1.34 %)
Diabetes Mellitus	106013 (4.73 %)	105639 (4.71 %)
Hypertension	204221 (9.11 %)	203969 (9.10 %)
Ischemic Heart Disease	21457 (0.96 %)	20448 (0.91 %)
Low back pain	171107 (7.63 %)	163786 (7.31 %)
Osteoporosis	19738 (0.88 %)	19411 (0.87 %)
Parkinson's disease	4551 (0.20 %)	4234 (0.19 %)
Persistent asthma	65269 (2.91 %)	63816 (2.85 %)
Rheumatoid arthritis	5342 (0.24 %)	5261 (0.23 %)
Schizophrenia & affective dis.	7593 (0.34 %)	7112 (0.32 %)
Seizure disorders	9149 (0.41 %)	8597 (0.38 %)

Table 1: Comparison between development and validation data. (COPD: Chronic Obstructive Pulmonary Disease). The comorbidity data indicates the number of patients with active diagnoses in the first year of study, i.e., those that had any related treatment, diagnostic code or medical appointment in 2016 (development) or 2017 (validation). The true prevalences of diseases may be higher.

Performance in the training set

See Table 2 and Figure 1.

Table 2: For the training set: Summary of the performance metrics in each family of models (quartiles and standard deviation). (MLP: Multi-Layer Perceptron; GBDT: Gradient-Boosting Decision Trees, LR: Logistic Regression, RF: Random Forest, AP: Average Precision, R@20k and PPV@20k: Recall and Positive Predictive Value for the 20000 highest-risk patients)

	25%	50%	75%	std
**AUC**				
MLP	0.8043	0.8045	0.8046	3.25e-04
GBDT	0.8040	0.8044	0.8059	1.41e-03
LR		0.7993		
RF	0.7839	0.7892	0.7894	4.25e-03
**AP**				
MLP	0.2606	0.2610	0.2613	1.13e-03
GBDT	0.2582	0.2593	0.2618	3.12e-03
LR		0.2490		
RF	0.2278	0.2421	0.2448	1.13e-02
**R@20k**				
MLP	0.0847	0.0850	0.0853	5.14e-04
GBDT	0.0840	0.0844	0.0854	1.13e-03
RF	0.0760	0.0817	0.0831	4.36e-03
LR		0.0808		
**PPV@20k**				
MLP	0.5243	0.5258	0.5277	3.18e-03
GBDT	0.5196	0.5222	0.5285	7.02e-03
RF	0.4704	0.5055	0.5143	2.70e-02
LR		0.5000		
**Brier Score**				
MLP	0.0461	0.0461	0.0461	3.51e-05
GBDT	0.0460	0.0461	0.0462	1.09e-04
LR		0.0465		
RF	0.0468	0.0468	0.0473	3.55e-04

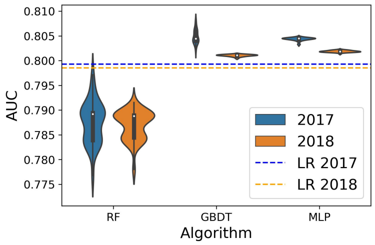

(a) AUC

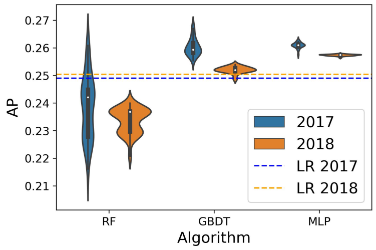

(b) AP

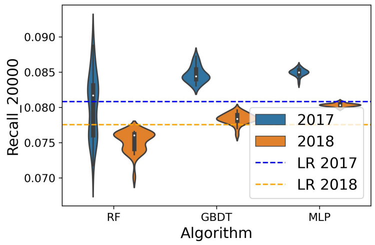

(c) R@20k

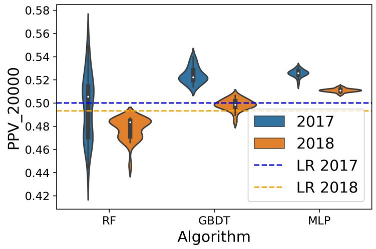

(d) PPV@20k

Figure 1: Performance of the RF, GBDT and MLP families according to different discrimination metrics, both in the training (2017) and external validation (2018) sets. (MLP: Multi-Layer Perceptron; GBDT: Gradient-Boosting Decision Trees, LR: Logistic Regression, RF: Random Forest, AP: Average Precision, R@20k and PPV@20k: Recall and Positive Predictive Value for the 20000 highest-risk patients).

ACG System: Description and variables initially included the prediction models

The Johns Hopkins ACG System is a statistically valid, case-mix methodology to describe or predict a population's past or future healthcare utilization and costs. The ACG System provides a number of markers derived from a patient's diagnosis and pharmacy code history from all encounters during a 12-month period.

Main variable groups:

- Patterns of Morbidity - Adjusted Clinical Groups (ACGs): ACGs are a series of mutually exclusive, health status categories defined by morbidity, age, and sex. They are based on the premise that the level of resources necessary for delivering appropriate healthcare to a population is correlated with the illness burden of that population.

- Morbidity Types - Aggregated Diagnosis Groups (ADGs): Each diagnosis code (International Classification of Disease-ICD) is assigned to one or more of 32 diagnosis groups referred to as Aggregated Diagnosis Groups, or ADGs. Diagnosis codes within the same ADG are similar in terms of both clinical criteria and expected need for healthcare resources. Some ADGs have very high expected resource use and are labeled as Major ADGs.

- Resource Utilization Bands (RUBs): ACGs are collapsed according to a combination of concurrent relative resource use and number of comorbidities in the creation of RUBs. The software automatically assigns six RUB classes.

- Disease Markers - Expanded Diagnosis Clusters (EDCs): Diagnosis codes found in claims or encounter data are assigned to one of 282 EDCs. Diagnosis codes within an EDC share similar clinical characteristics and are expected to evoke similar types of diagnostic and therapeutic responses.

- Hospital Dominant Morbidity Types: Hospital dominant morbidity types are based on diagnoses that, when present, are associated with a markedly greater probability of hospitalization among affected patients in the next year.

- Frailty Conditions: The Frailty Flag is a dichotomous variable that indicates whether an enrollee over the age of 18 has a diagnosis falling within any one of 10 clusters that represent medical problems associated with frailty.

- Rx-Defined Morbidity Groups (Rx-MGs): Rx-MGs are a medication classification system according to medication's mechanism of action, clinical indications, and route of administration information (67 Rx-MG categories).

- Active Ingredient Count: An active ingredient count is calculated as the count of unique active ingredient/route of administration combinations encountered in the patient's drug claims.

Reference: ACG System Technical Reference Guide (Health Services Research & Development Center at Johns Hopkins University, Bloomberg School of Public Health: The Johns Hopkins ACG System Reference Guide. Version 11.1. Baltimore, MD; 2016)

Predictive capacity of different groups of variables

See Table 3.

Model	AUC	AP	R@20k	PPV@20k	Brier Score
Age + sex	0.7318	0.1488	0.0532	0.2513	0.0507
Age + sex + ACG	0.7878	0.2148	0.0653	0.4071	0.0487
Age + sex + ACG + EDC	0.7954	0.2448	0.0761	0.4838	0.0479
Full model	0.7985	0.2504	0.0776	0.4932	0.0477

Table 3: For the external validation set: Predictive capacity of nested logistic regression models. The full model includes age, sex, Adjusted Clinical Groups (ACGs), Expanded Diagnostic Clusters (EDCs), and pharmacy-based markers (Rx-MGs). See additional file for the full list of variables. (AP: Average Precision, R@20k and PPV@20k: Recall and Positive Predictive Value for the 20000 highest-risk patients)

Variable importance

Figure 2 shows the 20 most important variables for the models with median R@20k.

In the case of logistic regression (Figure 2a) we examine the model coefficients. We see that being younger than 54 lowers the risk of hospitalization significantly (the reference category for age is being older than 85). Among the most influential risk factors, we highlight ACG 5030 (10+ Other ADG Combinations, Age 1-17, 2+ Major ADGs).

For the random forest model, we calculate the mean decrease of impurity linked to each variable (Figure 2b). The most influential variable is age (adding up the contributions of each category), followed by several pharmacy markers (CARX030: Cardiovascular/High Blood Pressure , GASX060: Gastrointestinal/Hepatic/Peptic Disease ; CARX050: Cardiovascular/Vascular Disorders...).

For GBDT and MLP, we compute SHAP (SHapley Additive exPlanations; Lundberg and Lee [2017], Lundberg et al. [2018]) values in a sample of 1000

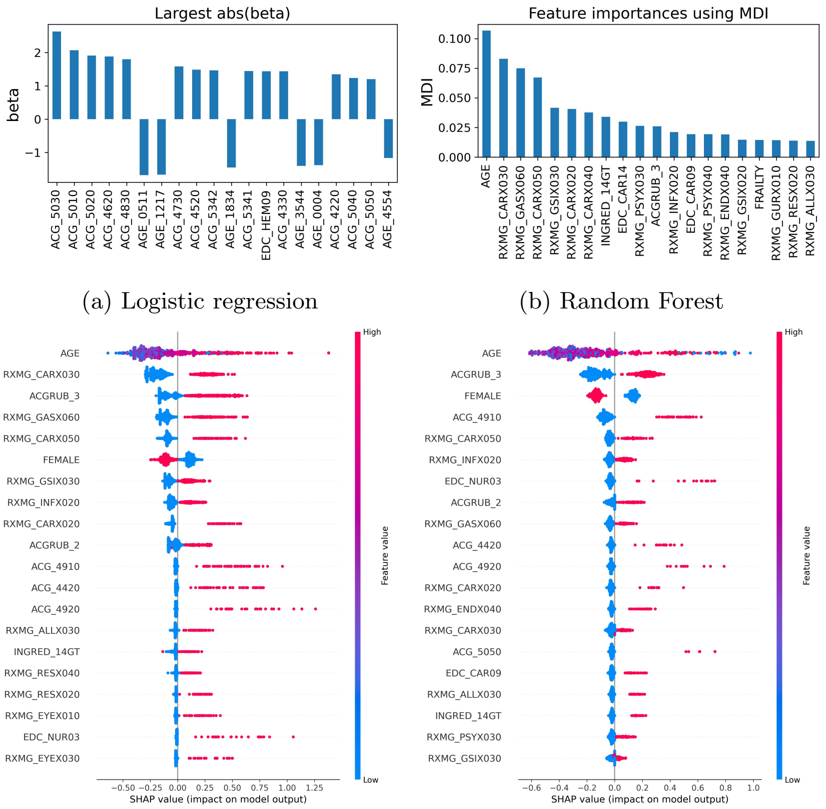

(a) Logistic regression

(b) Random Forest

(c) GBDT

(d) MLP

Figure 2: Variable importance for the models with median R@20k. See additional file for the full variable names. MDI: Mean Decrease in Impurity

and 500 patients, respectively, due to computational resource availability. In both cases, age is the most important variable, and the colored dots show that being older is correlated with higher predicted risk (positive impact on model output), but that the relationship is not straightforward. We can see this by noting that there are some young individuals with high SHAP values. In both models, being female decreases the predicted risk, and having a high resource utilization increases it (ACGRUB 2 and 3).

There is an overlap between the most important variables in different algorithms, mostly between the tree ensembles and MLP. These three methods appear to rely in pharmacy information and selected ACG categories, whereas logistic regression relies heavily on the ACGs. Age is an important variable in all cases.

Other decision thresholds

In the main text, we show results for the highest scoring 20000 patients (around 1%). Here, for completeness, we include results considering the top 5% and the top 10% as positive (likely to be hospitalized). See Table 4.

Table 4: For the external validation set: Summary of the performance metrics in each family of models (quantiles and standard deviation). The techniques are ordered from the best to the worst performance. (MLP: Multi-Layer Perceptron; GBDT: Gradient-Boosting Decision Trees, LR: Logistic Regression, RF: Random Forest, R@5% and PPV@5%; R@10% and PPV@10%: Recall and Positive Predictive Value for the 5% and 10% highest-risk patients)

	25%	50%	75%	std
**R@5%**				
MLP	0.2856	0.2858	0.2861	4.20e-04
GBDT	0.2817	0.2821	0.2828	7.64e-04
LR		0.2815		
RF	0.2590	0.2651	0.2655	4.02e-03
**PPV@5%**				
MLP	0.3242	0.3244	0.3248	4.76e-04
GBDT	0.3197	0.3201	0.3210	8.67e-04
LR		0.3195		
RF	0.2940	0.3009	0.3013	4.56e-03
**R@10%**				
MLP	0.4377	0.4380	0.4384	5.41e-04
GBDT	0.4349	0.4353	0.4355	6.60e-04
LR		0.4340		
RF	0.4042	0.4135	0.4136	5.72e-03
**PPV@10%**				
MLP	0.2484	0.2486	0.2488	3.07e-04
GBDT	0.2468	0.2470	0.2472	3.75e-04
LR		0.2463		
RF	0.2294	0.2346	0.2347	3.25e-03

References

S. M. Lundberg and S.-I. Lee. A unified approach to interpreting model predictions. In *Proceedings of the 31st International Conference on Neural Information Processing Systems*, NIPS'17, page 4768–4777, Red Hook, NY, USA, 2017. Curran Associates Inc. ISBN 9781510860964.

S. M. Lundberg, B. Nair, M. S. Vavilala, M. Horibe, M. J. Eisses, T. Adams, D. E. Liston, D. K.-W. Low, S.-F. Newman, J. Kim, et al. Explainable machine-learning predictions for the prevention of hypoxaemia during surgery. *Nature Biomedical Engineering*, 2(10):749, 2018.
