# Supplementary figures and images for: Development and validation of predictive models for unplanned hospitalization in the Basque Country: analyzing the variability of non-deterministic algorithms
Source: BMC Med Inform Decis Mak. 2023 Aug 5;23:152. doi: 10.1186/s12911-023-02226-z (PMC10403913; doi:10.1186/s12911-023-02226-z)

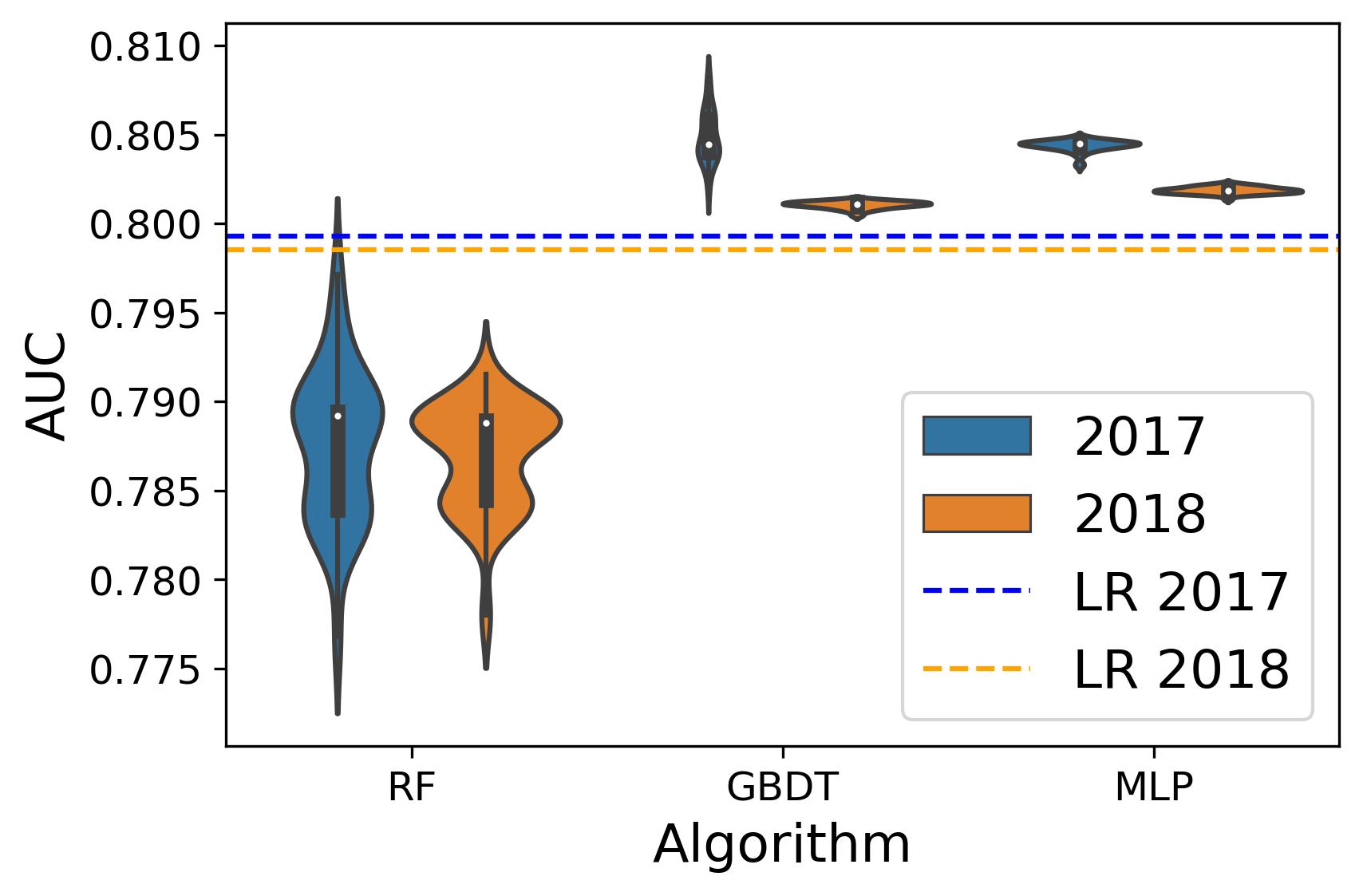

Supplement: Supplementary file 1 — Additional file 1. Appendix. [file 12911_2023_2226_MOESM1_ESM.zip › supplementary_figure1a.jpeg]

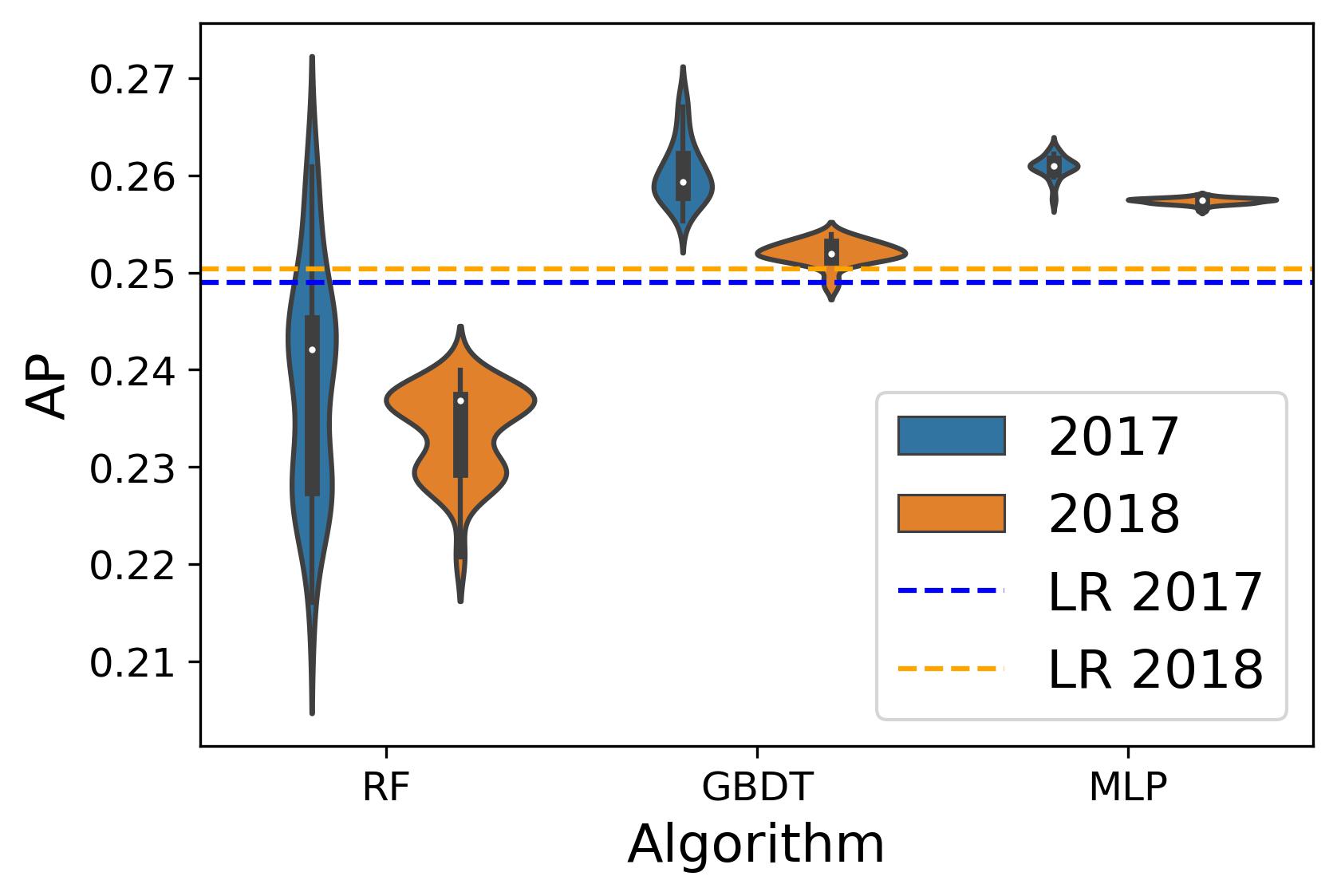

Supplement: Supplementary file 1 — Additional file 1. Appendix. [file 12911_2023_2226_MOESM1_ESM.zip › supplementary_figure1b.jpeg]

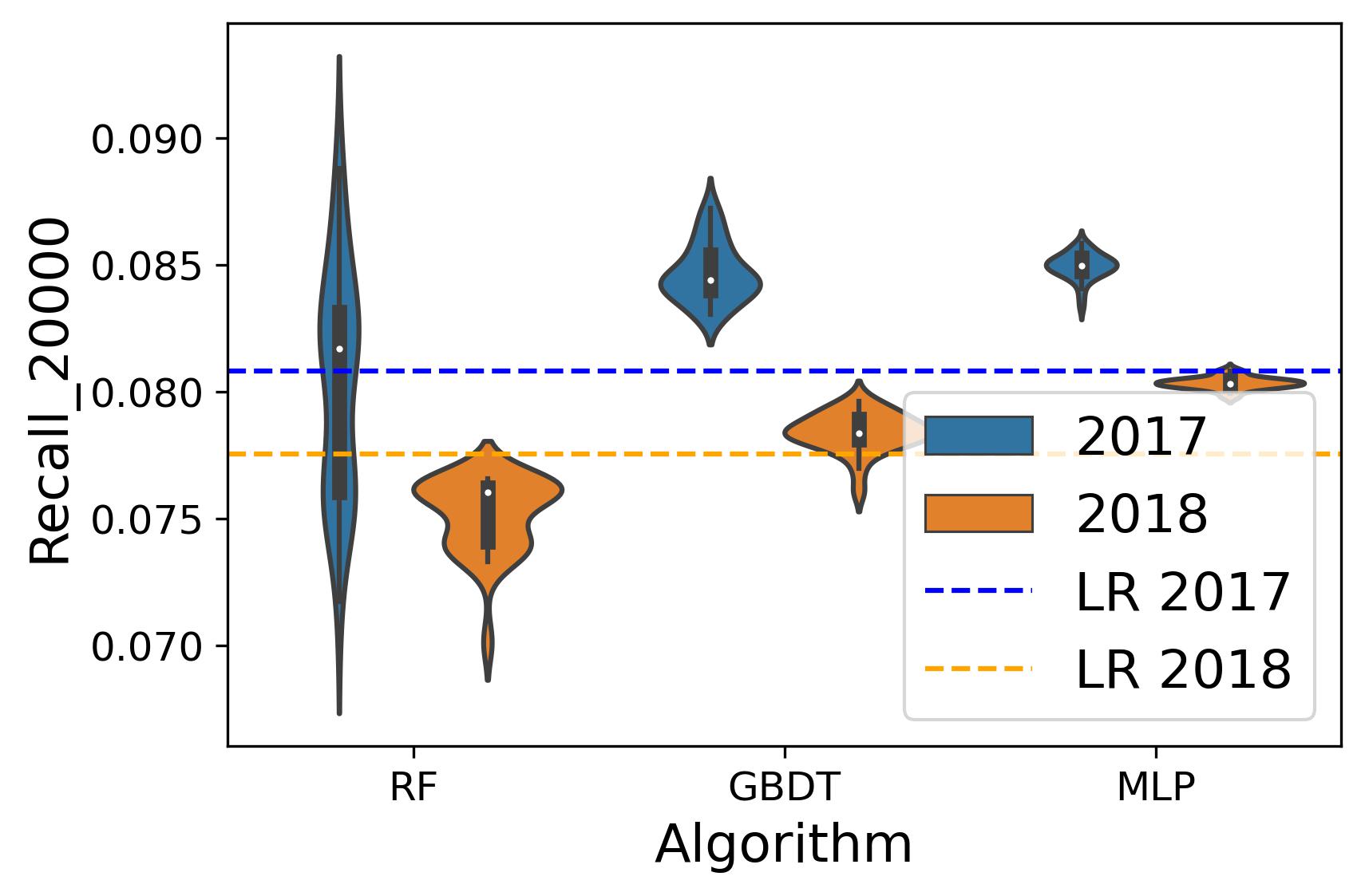

Supplement: Supplementary file 1 — Additional file 1. Appendix. [file 12911_2023_2226_MOESM1_ESM.zip › supplementary_figure1c.jpeg]

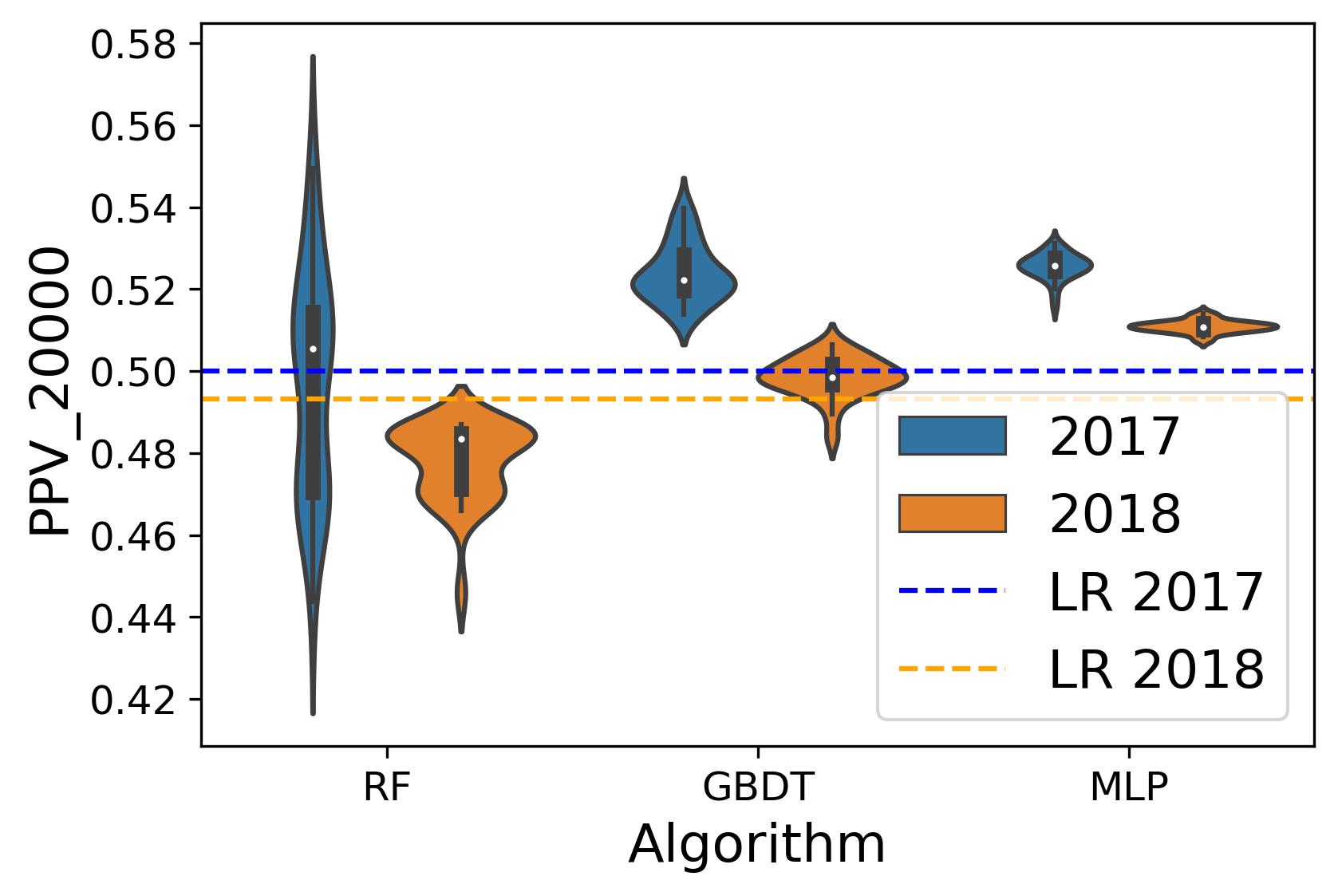

Supplement: Supplementary file 1 — Additional file 1. Appendix. [file 12911_2023_2226_MOESM1_ESM.zip › supplementary_figure1d.jpeg]

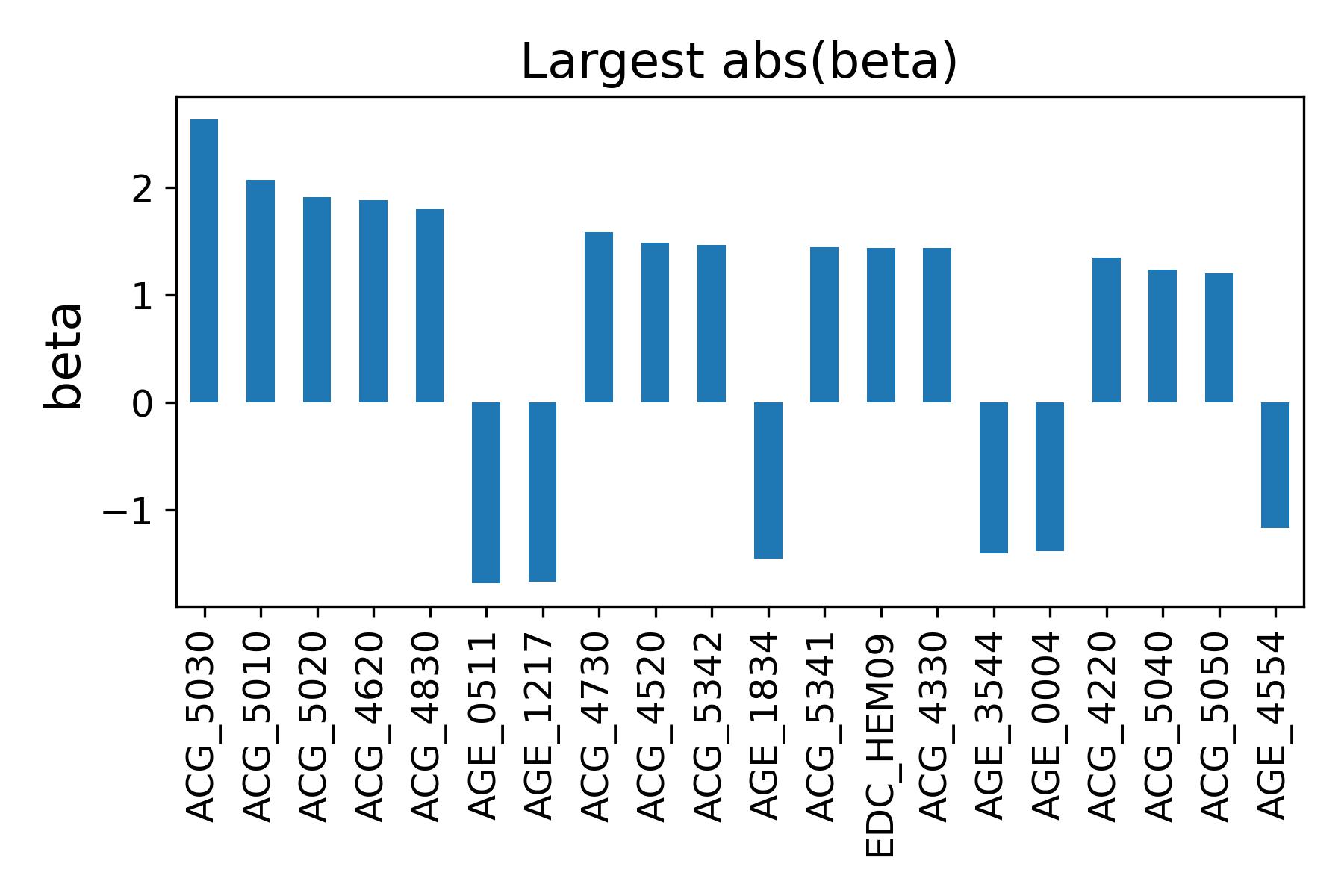

Supplement: Supplementary file 1 — Additional file 1. Appendix. [file 12911_2023_2226_MOESM1_ESM.zip › supplementary_figure2a.jpeg]

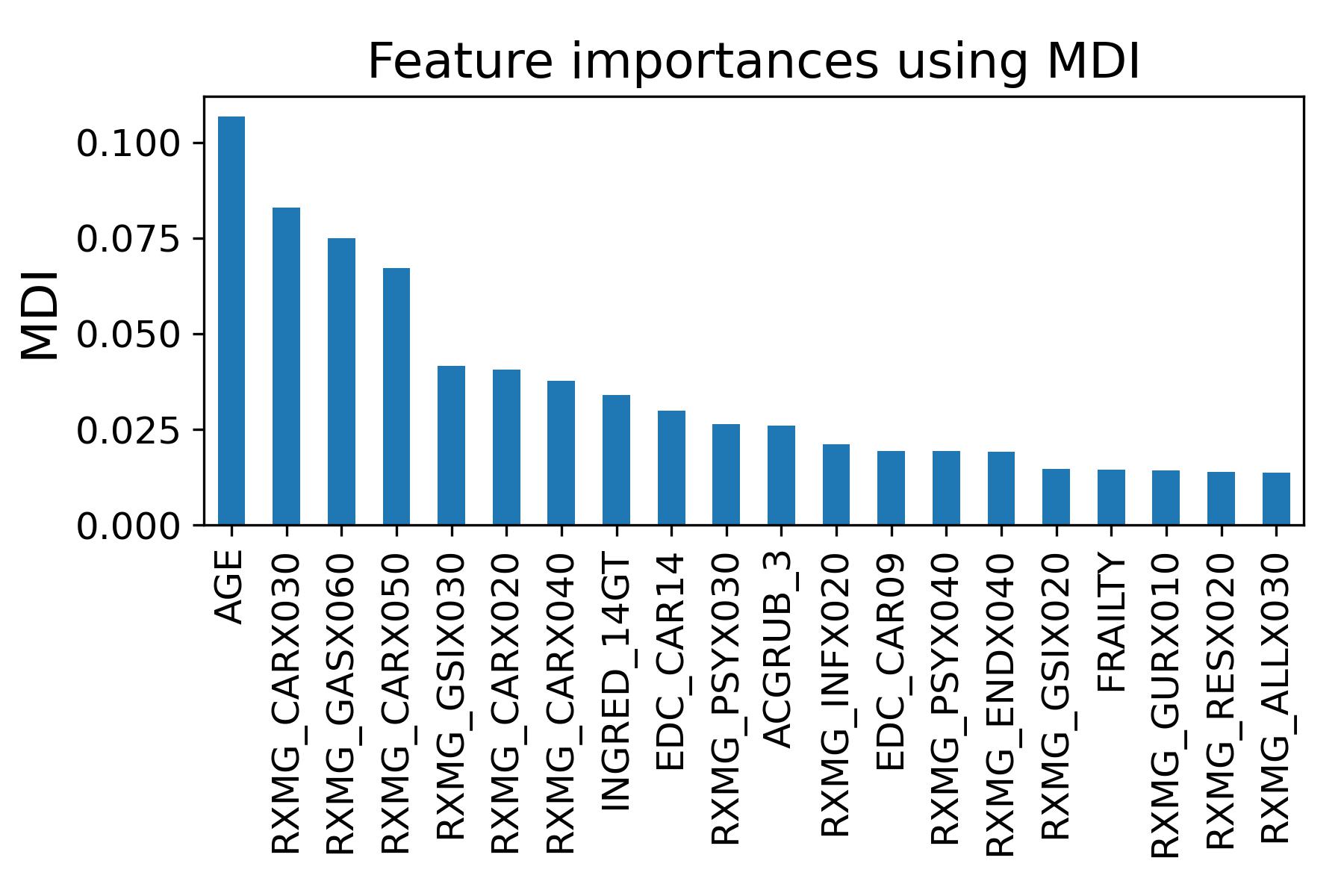

Supplement: Supplementary file 1 — Additional file 1. Appendix. [file 12911_2023_2226_MOESM1_ESM.zip › supplementary_figure2b.jpeg]

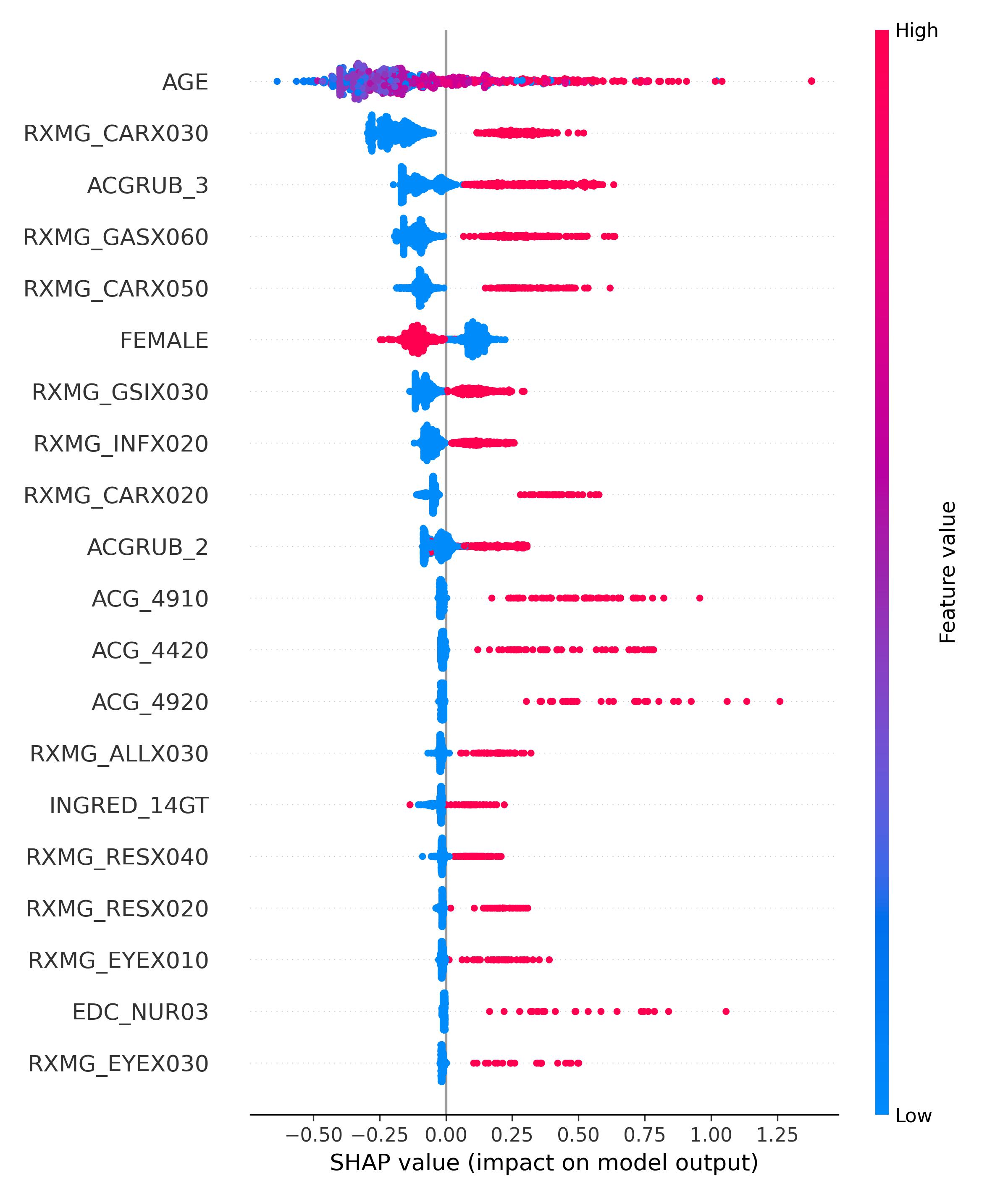

Supplement: Supplementary file 1 — Additional file 1. Appendix. [file 12911_2023_2226_MOESM1_ESM.zip › supplementary_figure2c.jpeg]

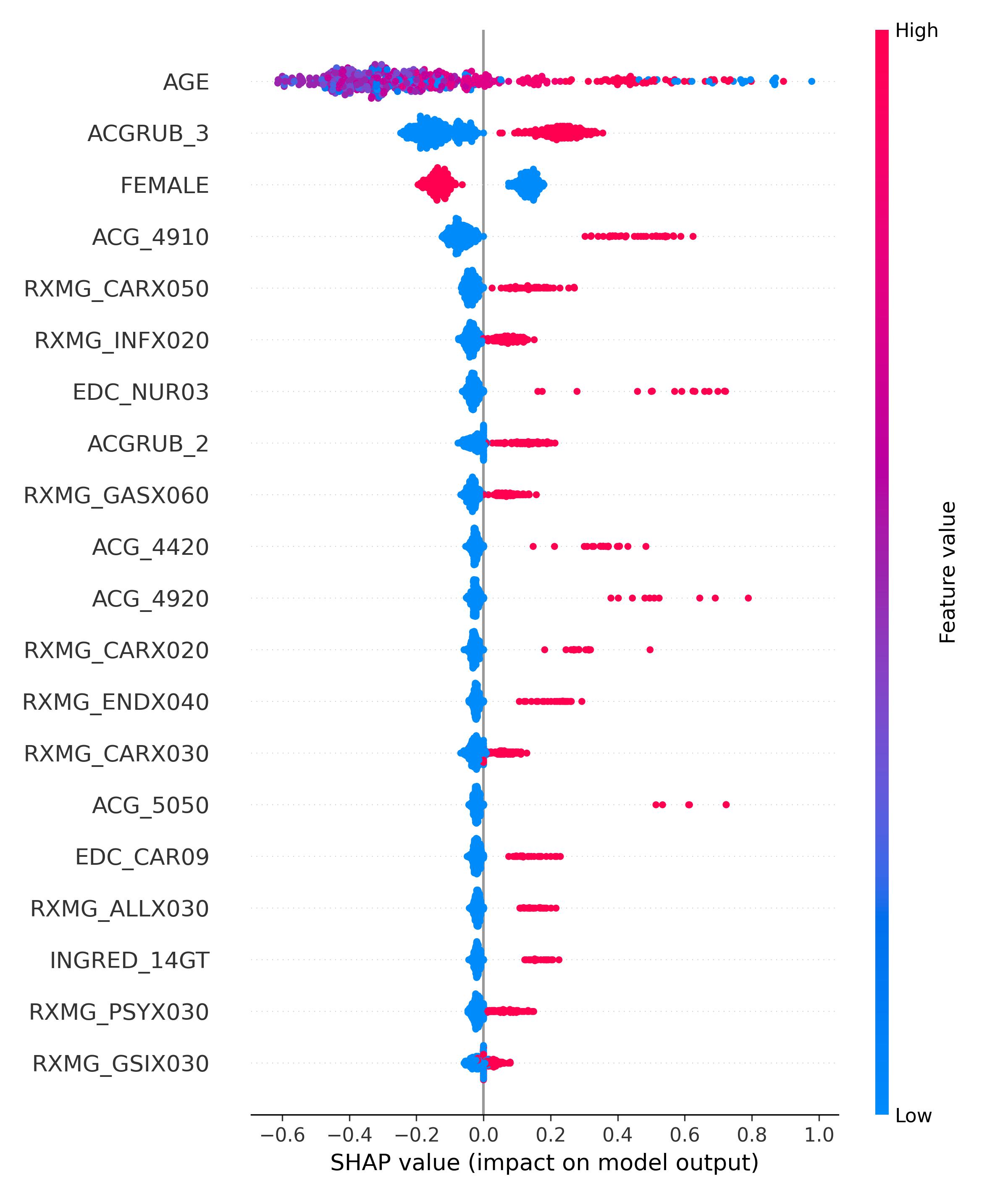

Supplement: Supplementary file 1 — Additional file 1. Appendix. [file 12911_2023_2226_MOESM1_ESM.zip › supplementary_figure2d.jpeg]
