# Supplementary material for: Development and validation of predictive models for unplanned hospitalization in the Basque Country: analyzing the variability of non-deterministic algorithms
Source: BMC Med Inform Decis Mak. 2023 Aug 5;23:152. doi: 10.1186/s12911-023-02226-z (PMC10403913; doi:10.1186/s12911-023-02226-z)
Supplement: Supplementary file 2 — Additional file 2. Full list of variables included in the models and TRIPOD checklist: Good practices for prediction model development and validation. [file 12911_2023_2226_MOESM2_ESM.pdf]

Predictor variables included in the models (ADDITIONAL FILE)

Reference: ACG System Technical Reference Guide (Health Services Research & Development Center at Johns Hopkins University, Bloomberg School of Public Health: The Johns Hopkins ACG System Reference Guide. Version 11.1. Baltimore, MD; 2016)

- ACG:
  - 4220 4-5 Other ADG Combinations, Age 1-17, 1+ Major ADGs
  - 4330 4-5 Other ADG Combinations, Age 18-44, 2+ Major ADGs
  - 4420 4-5 Other ADG Combinations, Age ¿44, 1 Major ADGs
  - 4430 4-5 Other ADG Combinations, Age ¿44, 2+ Major ADGs
  - 4510 6-9 Other ADG Combinations, Age 1-5, No Major ADGs
  - 4520 6-9 Other ADG Combinations, Age 1-5, 1+ Major ADGs
  - 4610 6-9 Other ADG Combinations, Age 6-17, No Major ADGs
  - 4620 6-9 Other ADG Combinations, Age 6-17, 1+ Major ADGs
  - 4730 6-9 Other ADG Combinations, Male, Age 18-34, 2+ Major ADGs
  - 4830 6-9 Other ADG Combinations, Female, Age 18-34, 2+ Major ADGs
  - 4910 6-9 Other ADG Combinations, Age ¿34, 0-1 Major ADGs
  - 4920 6-9 Other ADG Combinations, Age ¿34, 2 Major ADGs
  - 4930 6-9 Other ADG Combinations, Age ¿34, 3 Major ADGs
  - 4940 6-9 Other ADG Combinations, Age ¿34, 4+ Major ADGs
  - 5010 10+ Other ADG Combinations, Age 1-17 No Major ADGs
  - 5020 10+ Other ADG Combinations, Age 1-17, 1 Major ADGs
  - 5030 10+ Other ADG Combinations, Age 1-17, 2+ Major ADGs
  - 5040 10+ Other ADG Combinations, Age 18+, 0-1 Major ADGs
  - 5050 10+ Other ADG Combinations, Age 18+, 2 Major ADGs
  - 5060 10+ Other ADG Combinations, Age 18+, 3 Major ADGs
  - 5070 10+ Other ADG Combinations, Age 18+, 4+ Major ADGs
  - 5320 Infants: 0-5 ADGs, 1+ Major ADGs
  - 5321 Infants: 0-5 ADGs, 1+ Major ADGs, low birth weight
  - 5322 Infants: 0-5 ADGs, 1+ Major ADGs, normal birth weight
  - 5330 Infants: 6+ ADGs, No Major ADGs
  - 5331 Infants: 6+ ADGs, No Major ADGs, low birth weight
  - 5332 Infants: 6+ ADGs, No Major ADGs, normal birth weight
  - 5340 Infants: 6+ ADGs, 1+ Major ADG
  - 5341 Infants: 6+ ADGs, 1+ Major ADG, low birth weight
  - 5342 Infants: 6+ ADGs, 1+ Major ADG, normal birth weight
  - PREGNANCY
- RUB:
  - RUB1
  - RUB2
  - RUB3
- EDC
  - ADM03 Transplant Status
  - ALL04 Asthma, w/o status asthmaticus
  - ALL05 Asthma, with status asthmaticus
  - ALL06 Disorders of the Immune System

- CAR03 Ischemic heart disease (excluding acute myocardial infarction)
- CAR04 Congenital heart disease
- CAR05 Congestive heart failure
- CAR06 Cardiac valve disorders
- CAR07 Cardiomyopathy
- CAR09 Cardiac arrhythmia
- CAR10 Generalized atherosclerosis
- CAR12 Acute Myocardial Infarction
- CAR13 Cardiac arrest, shock
- CAR14 Hypertension, w/o major complications
- CAR15 Hypertension, with major complications
- END02 Osteoporosis
- END06 Type 2 Diabetes, w/o Complication
- END07 Type 2 Diabetes, w/ Complication
- END08 Type 1 Diabetes, w/o Complication
- END09 Type 1 Diabetes, w/ Complication
- EYE03 Retinal disorders (excluding diabetic retinopathy)
- EYE13 Diabetic Retinopathy
- EYE15 Age-related Macular Degeneration
- FRE03 Endometriosis
- FRE05 Female Infertility
- FRE12 Utero-vaginal prolapse
- GAS02 Inflammatory bowel disease
- GAS04 Acute hepatitis
- GAS05 Chronic liver disease
- GAS06 Peptic ulcer disease
- GAS10 Diverticular disease of colon
- GAS11 Acute pancreatitis
- GAS12 Chronic pancreatitis
- GSI08 Edema
- GSU11 Peripheral vascular disease
- GSU13 Aortic aneurysm
- GSU14 Gastrointestinal Obstruction/Perforation
- GTC01 Chromosomal anomalies
- GUR04 Prostatic hypertrophy
- HEM01 Other hemolytic anemias
- HEM03 Thrombophlebitis
- HEM05 Aplastic anemia
- HEM06 Deep vein thrombosis
- HEM07 Hemophilia, coagulation Disorder
- HEM09 Sickle Cell Disease
- INF04 HIV, AIDS
- INF08 Septicemia
- MAL02 Low impact malignant neoplasms
- MAL03 High impact malignant neoplasms
- MAL04 Malignant neoplasms, breast

- MAL06 Malignant neoplasms, ovary
- MAL07 Malignant neoplasms, esophagus
- MAL08 Malignant neoplasms, kidney
- MAL09 Malignant neoplasms, liver and biliary tract
- MAL10 Malignant neoplasms, lung
- MAL11 Malignant neoplasms, lymphomas
- MAL12 Malignant neoplasms, colorectal
- MAL13 Malignant neoplasms, pancreas
- MAL14 Malignant neoplasms, prostate
- MAL15 Malignant neoplasms, stomach
- MAL16 Acute Leukemia
- MAL18 Malignant neoplasms, bladder
- MUS03 Degenerative joint disease
- MUS10 Fracture of neck of femur (hip)
- MUS14 Low back pain
- MUS16 Amputation Status
- NUR03 Peripheral neuropathy, neuritis
- NUR05 Cerebrovascular disease
- NUR06 Parkinson's disease
- NUR07 Seizure disorder
- NUR08 Multiple sclerosis
- NUR09 Muscular dystrophy
- NUR12 Quadriplegia and Paraplegia
- NUR15 Head Injury
- NUR16 Spinal Cord Injury/Disorders
- NUR17 Paralytic Syndromes, Other
- NUR18 Cerebral Palsy
- NUR19 Developmental disorder
- NUR23 Organic Brain Syndrome
- NUR24 Dementia
- NUR25 Delirium
- NUR26 Autism Spectrum Disorders
- NUT02 Nutritional deficiencies
- PSY01 Anxiety, neuroses
- PSY02 Substance use
- PSY03 Tobacco abuse
- PSY05 Attention deficit disorder
- PSY07 Schizophrenia and affective psychosis
- PSY08 Personality disorders
- PSY09 Depression
- PSY12 Bipolar Disorder
- PSY13 Adjustment disorder
- PSY14 Psychosocial disorders of childhood
- PSY15 Eating disorder
- PSY16 Impulse control
- PSY17 Psycho-physiologic and somatoform disorders

  - PSY18 Psychosexual
  - PSY19 Sleep disorders of nonorganic origin
  - PSY20 Major depression
  - REC01 Cleft lip and palate
  - REC03 Chronic ulcer of the skin
  - REC04 Burns–2nd and 3rd degree
  - REN01 Chronic renal failure
  - REN02 Fluid/electrolyte disturbances
  - REN03 Acute renal failure
  - REN04 Nephritis/Nephrosis
  - REN06 End stage renal disease
  - RES02 Acute lower respiratory tract infection
  - RES03 Cystic fibrosis
  - RES04 Emphysema, chronic bronchitis, COPD
  - RES08 Pulmonary embolism
  - RES09 Tracheostomy
  - RES12 Acute respiratory failure
  - RES13 Chronic respiratory failure
  - RES14 Aspiration and bacterial pneumonias
  - RHU01 Autoimmune and connective tissue diseases
  - RHU05 Rheumatoid Arthritis
  - TOX02 Adverse effects of medicinal agents
  - TOX04 Complications of Mechanical Devices
- Rx-MGs
  - ALLx010 Allergy/Immunology/Acute Minor
  - ALLx030 Allergy/Immunology/Chronic Inflammatory
  - ALLx040 Allergy/Immunology/Immune Disorders
  - ALLx050 Allergy/Immunology/Transplant
  - CARx010 Cardiovascular/Chronic Medical
  - CARx020 Cardiovascular/Congestive Heart Failure
  - CARx030 Cardiovascular/High Blood Pressure
  - CARx040 Cardiovascular/Hyperlipidemia
  - CARx050 Cardiovascular/Vascular Disorders
  - EARx010 Ears, Nose, Throat/Acute Minor
  - ENDx010 Endocrine/Bone Disorders
  - ENDx020 Endocrine/Chronic Medical
  - ENDx030 Endocrine/Diabetes With Insulin
  - ENDx040 Endocrine/Diabetes Without Insulin
  - ENDx050 Endocrine/Thyroid Disorders
  - ENDx060 Endocrine/Growth Problems
  - ENDx070 Endocrine/Weight Control
  - EYEx010 Eye/Acute Minor: Curative
  - EYEx020 Eye/Acute Minor: Palliative
  - EYEx030 Eye/Glaucoma
  - FREx010 Female Reproductive/Hormone Regulation
  - FREx020 Female Reproductive/Infertility

  - FREx030 Female Reproductive/Pregnancy and Delivery
  - GASx010 Gastrointestinal/Hepatic/Acute Minor
  - GASx020 Gastrointestinal/Hepatic/Chronic Liver Disease
  - GASx030 Gastrointestinal/Hepatic/Chronic Stable
  - GASx040 Gastrointestinal/Hepatic/Inflammatory Bowel Disease
  - GASx050 Gastrointestinal/Hepatic/Pancreatic Disorder
  - GASx060 Gastrointestinal/Hepatic/Peptic Disease
  - GSIx010 General Signs and Symptoms/Nausea and Vomiting
  - GSIx020 General Signs and Symptoms/Pain
  - GSIx030 General Signs and Symptoms/Pain and Inflammation
  - GSIx040 General Signs and Symptons/Severe Pain
  - GURx010 Genito-Urinary/Acute Minor
  - GURx020 Genito-Urinary/Chronic Renal Failure
  - HEMx010 Hematologic/Coagulation Disorders
  - INFx010 Infections/Acute Major
  - INFx020 Infections/Acute Minor
  - INFx030 Infections/HIV/AIDS
  - INFx040 Infections/Tuberculosis
  - INFx050 Infections/Severe Acute Major
  - MALx010 Malignancies
  - MUSx010 Musculoskeletal/Gout
  - MUSx020 Musculoskeletal/Inflammatory Conditions
  - NURx010 Neurologic/Alzheimer's Disease
  - NURx020 Neurologic/Chronic Medical
  - NURx030 Neurologic/Migraine Headache
  - NURx040 Neurologic/Parkinsons Disease
  - NURx050 Neurologic/Seizure Disorder
  - PSYx010 Psychosocial/Attention Deficit Hyperactivity Disorder
  - PSYx020 Psychosocial/Addiction
  - PSYx030 Psychosocial/Anxiety
  - PSYx040 Psychosocial/Depression
  - PSYx050 Psychosocial/Acute Minor
  - PSYx060 Psychosocial/Chronic Unstable
  - PSYx070 Psychosocial/Sleep Disorders
  - PSYx080 Psychosocial/Tobacco Cessation
  - PSYx090 Psychosocial/Bipolar Disorder
  - RESx010 Respiratory/Acute Minor
  - RESx020 Respiratory/Chronic Medical
  - RESx030 Respiratory/Cystic Fibrosis
  - RESx040 Respiratory/Airway Hyperactivity
  - SKNx010 Skin/Acne
  - SKNx020 Skin/Acute and Recurrent
  - SKNx030 Skin/Chronic Medical
  - TOXx010 Toxic Effects/Adverse Effects/Acute Major
- Frailty Marker
- Active Ingredient Count - Boolean indicator for 14 or more Active Ingredients

- Demographic markers - Age 0-4, 5-11, 12-17, 18-34, 35-44, 45-54, 55-69,70-74, 75-79, 80-84, 85+ ; FEMALE

TRIPOD Checklist: Prediction Model Development and Validation

Section/Topic	Item		Checklist Item	Page
**Title and abstract**				
Title	1	D;V	Identify the study as developing and/or validating a multivariable prediction model, the target population, and the outcome to be predicted.	1
Abstract	2	D;V	Provide a summary of objectives, study design, setting, participants, sample size, predictors, outcome, statistical analysis, results, and conclusions.	1
**Introduction**				
Background and objectives	3a	D;V	Explain the medical context (including whether diagnostic or prognostic) and rationale for developing or validating the multivariable prediction model, including references to existing models.	1,2
	3b	D;V	Specify the objectives, including whether the study describes the development or validation of the model or both.	2
**Methods**				
Source of data	4a	D;V	Describe the study design or source of data (e.g., randomized trial, cohort, or registry data), separately for the development and validation data sets, if applicable.	3
	4b	D;V	Specify the key study dates, including start of accrual; end of accrual; and, if applicable, end of follow-up.	3
Participants	5a	D;V	Specify key elements of the study setting (e.g., primary care, secondary care, general population) including number and location of centres.	3
	5b	D;V	Describe eligibility criteria for participants.	3
	5c	D;V	Give details of treatments received, if relevant.	No treatments
Outcome	6a	D;V	Clearly define the outcome that is predicted by the prediction model, including how and when assessed.	3
	6b	D;V	Report any actions to blind assessment of the outcome to be predicted.	No action
Predictors	7a	D;V	Clearly define all predictors used in developing or validating the multivariable prediction model, including how and when they were measured.	3, addit. file 1
	7b	D;V	Report any actions to blind assessment of predictors for the outcome and other predictors.	n.a.
Sample size	8	D;V	Explain how the study size was arrived at.	3
Missing data	9	D;V	Describe how missing data were handled (e.g., complete-case analysis, single imputation, multiple imputation) with details of any imputation method.	No missing data
Statistical analysis methods	10a	D	Describe how predictors were handled in the analyses.	3
	10b	D	Specify type of model, all model-building procedures (including any predictor selection), and method for internal validation.	4
	10c	V	For validation, describe how the predictions were calculated.	4
	10d	D;V	Specify all measures used to assess model performance and, if relevant, to compare multiple models.	5,6
	10e	V	Describe any model updating (e.g., recalibration) arising from the validation, if done.	5
Risk groups	11	D;V	Provide details on how risk groups were created, if done.	6
Development vs. validation	12	V	For validation, identify any differences from the development data in setting, eligibility criteria, outcome, and predictors.	3, 7
**Results**				
Participants	13a	D;V	Describe the flow of participants through the study, including the number of participants with and without the outcome and, if applicable, a summary of the follow-up time. A diagram may be helpful.	Table 1
	13b	D;V	Describe the characteristics of the participants (basic demographics, clinical features, available predictors), including the number of participants with missing data for predictors and outcome.	Table 1
	13c	V	For validation, show a comparison with the development data of the distribution of important variables (demographics, predictors and outcome).	Appendix
Model development	14a	D	Specify the number of participants and outcome events in each analysis.	Table 1
	14b	D	If done, report the unadjusted association between each candidate predictor and outcome.	Not done
Model specification	15a	D	Present the full prediction model to allow predictions for individuals (i.e., all regression coefficients, and model intercept or baseline survival at a given time point).	Several models
	15b	D	Explain how to the use the prediction model.	Not delivered to the public
Model performance	16	D;V	Report performance measures (with CIs) for the prediction model.	Table 2
Model-updating	17	V	If done, report the results from any model updating (i.e., model specification, model performance).	No updating
**Discussion**				
Limitations	18	D;V	Discuss any limitations of the study (such as nonrepresentative sample, few events per predictor, missing data).	14
Interpretation	19a	V	For validation, discuss the results with reference to performance in the development data, and any other validation data.	Appendix
	19b	D;V	Give an overall interpretation of the results, considering objectives, limitations, results from similar studies, and other relevant evidence.	13,14
Implications	20	D;V	Discuss the potential clinical use of the model and implications for future research.	13
**Other information**				
Supplementary information	21	D;V	Provide information about the availability of supplementary resources, such as study protocol, Web calculator, and data sets.	18
Funding	22	D;V	Give the source of funding and the role of the funders for the present study.	18

*Items relevant only to the development of a prediction model are denoted by D, items relating solely to a validation of a prediction model are denoted by V, and items relating to both are denoted D;V. We recommend using the TRIPOD Checklist in conjunction with the TRIPOD Explanation and Elaboration document.
